# Targeting cell death pathways for cancer therapy: recent developments in necroptosis, pyroptosis, ferroptosis, and cuproptosis research

**DOI:** 10.1186/s13045-022-01392-3

**Published:** 2022-12-08

**Authors:** Xuhui Tong, Rong Tang, Mingming Xiao, Jin Xu, Wei Wang, Bo Zhang, Jiang Liu, Xianjun Yu, Si Shi

**Affiliations:** 1 Department of Pancreatic Surgery, Fudan University Shanghai Cancer Center, No. 270 Dong’An Road, Shanghai, 200032 China; 2 Department of Oncology, Shanghai Medical College, Fudan University, Shanghai, China; 3 Shanghai Pancreatic Cancer Institute, No. 270 Dong’An Road, Shanghai, 200032 China; 4 Pancreatic Cancer Institute, Fudan University, Shanghai, China

**Keywords:** Necroptosis, Pyroptosis, Ferroptosis, Cuproptosis, Tumor microenvironment, Nanoparticles

## Abstract

Many types of human cells self-destruct to maintain biological homeostasis and defend the body against pathogenic substances. This process, called regulated cell death (RCD), is important for various biological activities, including the clearance of aberrant cells. Thus, RCD pathways represented by apoptosis have increased in importance as a target for the development of cancer medications in recent years. However, because tumor cells show avoidance to apoptosis, which causes treatment resistance and recurrence, numerous studies have been devoted to alternative cancer cell mortality processes, namely necroptosis, pyroptosis, ferroptosis, and cuproptosis; these RCD modalities have been extensively studied and shown to be crucial to cancer therapy effectiveness. Furthermore, evidence suggests that tumor cells undergoing regulated death may alter the immunogenicity of the tumor microenvironment (TME) to some extent, rendering it more suitable for inhibiting cancer progression and metastasis. In addition, other types of cells and components in the TME undergo the abovementioned forms of death and induce immune attacks on tumor cells, resulting in enhanced antitumor responses. Hence, this review discusses the molecular processes and features of necroptosis, pyroptosis, ferroptosis, and cuproptosis and the effects of these novel RCD modalities on tumor cell proliferation and cancer metastasis. Importantly, it introduces the complex effects of novel forms of tumor cell death on the TME and the regulated death of other cells in the TME that affect tumor biology. It also summarizes the potential agents and nanoparticles that induce or inhibit novel RCD pathways and their therapeutic effects on cancer based on evidence from in vivo and in vitro studies and reports clinical trials in which RCD inducers have been evaluated as treatments for cancer patients. Lastly, we also summarized the impact of modulating the RCD processes on cancer drug resistance and the advantages of adding RCD modulators to cancer treatment over conventional treatments.

## Introduction

Cell death (especially cell suicide) plays a fundamental role in maintaining physiological homeostasis by removing damaged cells, and it may also be an aberrant pathological reaction to damaging stimuli [1]. The Nomenclature of Cell Death Committee has developed guidelines to divide cell death modes into accidental cell death and regulated cell death (RCD) according to morphology, biochemistry, and function [2]. Accidental cell death is a biologically uncontrolled process of cell death in response to accidental injury stimuli [3]. However, RCD is characterized by controlled signaling pathways that play key roles in organismal development or tissue renewal [4]. Previously, apoptosis was thought to be the major form of RCD, but with more in-depth study on tumor cell biology and thorough examination of cancer therapy mechanisms, more and more subtypes of RCD are progressively emerging. The novel RCD types we are going to introduce include: necroptosis, pyroptosis, ferroptosis, and cuproptosis, which can occur with or without exogenous environmental or intracellular perturbations [5–7]. Malignant cells, on the other hand, continue to evade the RCD routes through evolving a variety of mechanisms [8]. Additionally, RCD pathways have also been reported to be crucial for the prognosis of cancer patients, cancer progression and metastasis, and cancer immune surveillance [9–14]. Based on accumulating evidence, distinct forms of RCD might change the tumor microenvironment (TME) by releasing pathogen- or damage-associated molecular patterns (PAMPs or DAMPs), which affect the benefits of anticancer therapy [15–17].

Our review outlines the molecular mechanisms and processes of four different types of RCD, necroptosis, pyroptosis, ferroptosis, and the newly discovered cuproptosis, as well as their different roles in the initiation and progression of cancer. We specifically focus on RCD processes that influence the TME and the latest advancements in targeting necroptosis, pyroptosis, ferroptosis, and cuproptosis for cancer therapy. We describe the mechanisms of the various cancer therapies currently available, showing that they mainly depend on different RCD modalities. A reasonable assumption is that these novel RCD modalities constitute a mechanism of defense against tumor progression and migration. Furthermore, the significance and prevalence of RCDs in combating cancer drug resistance have been included, demonstrating that the use of conventional therapy in conjunction with RCD modulators might hold significant potential for cancer treatment. Hopefully, this information will lead to improved guidance for approaches to tumor therapy.

### Molecular mechanisms of different cell death pathways

The most extensively studied RCD modality is apoptosis, which leads to immunogenicity or induces no immunogenic response in different contexts [18, 19]. The morphological changes observed during apoptotic cell death include cell shrinkage, externalization of phosphatidylserine on the plasma membrane, and nuclear pyknosis and karyorrhexis; notably, the plasma membrane remains intact [2]. This pathway is believed to function as a natural barrier against malignancy, but the primary hallmark of cancer cells and the emergence of chemotherapy resistance during cancer therapy are limiting or causing cells to resist apoptosis [20, 21]. Therefore, while tackling apoptosis resistance, discover methods that induce nonapoptotic forms of RCD must be discovered as alternative cancer therapies. Excitingly, new forms of RCD have been extensively studied in the past decade; these modalities include necroptosis [22], pyroptosis [23], and ferroptosis [24]. Additionally, a noteworthy finding reported in 2022 is the description of cuproptosis, a previously unknown form of RCD [25].

#### Necroptosis

**Fig. 1:**
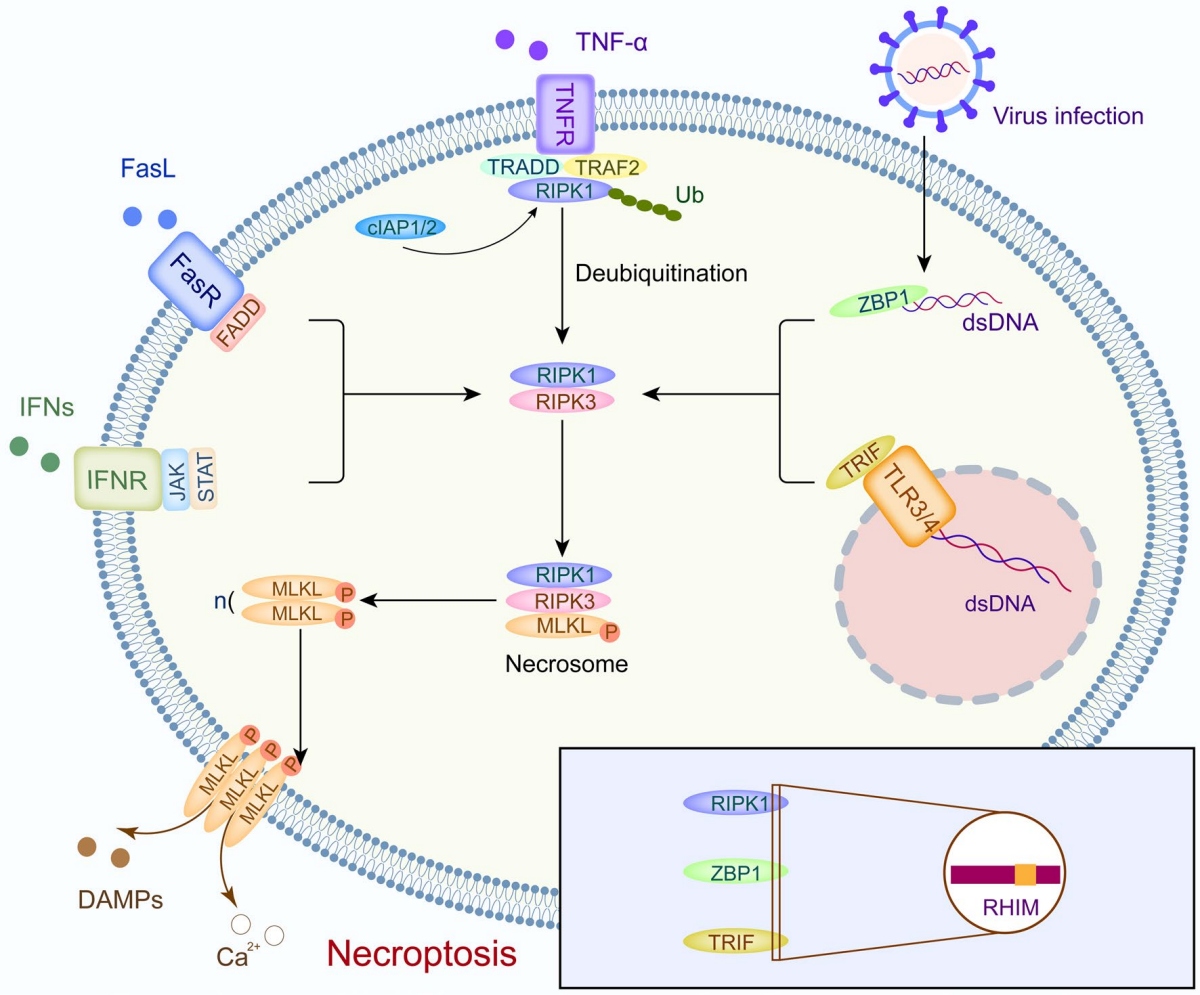
Detailed mechanism of necroptosis. Necroptosis is initiated by the cell surface death receptors (including FasRs, TNFR, IFN receptors, and TLRs) and ZBP1 in cells, and downstream proteins which contain RHIM bind to RIPK3. Subsequently, the necrosome is formed and led to cell lysis

Necroptosis is a regulated form of necrosis that depends on the phosphorylation of mixed-lineage kinase-like (MLKL) by receptor interacting kinase-1 (RIPK1) and RIPK3 [26]. The necroptotic process is initiated by the activation of cell surface death receptors (such as FasRs, TNFR1, IFN receptors, and TLRs) and RNA- and DNA-sensing molecules in cells. RIPK3 is required for the necroptotic process, and RIPK3 is activated by three known processes [27]. First, ligation of TNFR1 activates RIPK1, which in turn binds to RIPK3 via shared RIP homology interaction motifs (RHIM) present in both molecules [28, 29]. Similarly, engagement of TLR-3 and TLR-4 recruits the adapter, which contains an RHIM that is capable of binding and activating RIPK3 [27]. Finally, the cytosolic nucleic acid sensor Z-dsDNA/dsRNA-binding protein 1 (ZBP1) also contains a RIPK3-activating RHIM (Fig. 1) [30]. Subsequently, RIPK3 frequently phosphorylates MLKL, which oligomerizes to form an activated “necrosome” complex and is translocated to the plasma membrane. This process eventually leads to cell death characterized by permeabilization of the plasma membrane, cell swelling, and loss of cellular and organelle integrity [31, 32]. The rupture of plasma membrane results in cytokine, chemokine, and potassium efflux, leading to inflammation and immune responses [33]. Necroptosis has been pharmacologically suppressed using chemical compounds, including necrostatin-1 [34]. Recently, the significance of necroptosis in cancer has been increasingly appreciated, and a greater comprehension of necroptotic processes might be helpful in creating novel strategies for controlling cancer [9].

#### Pyroptosis

**Fig. 2:**
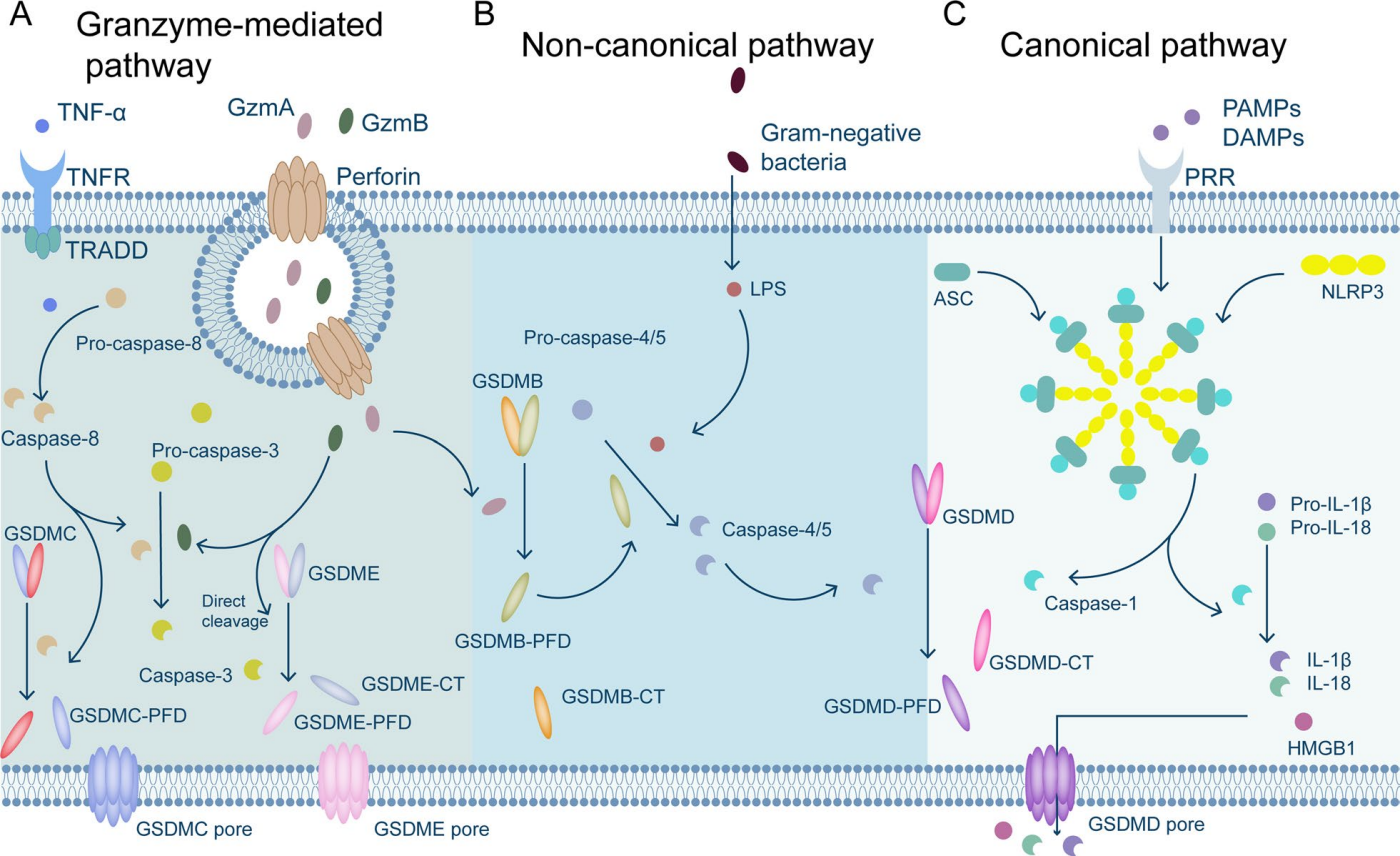
Summary of pyroptosis mediated by different cellular mechanisms. **A** Pyroptosis induced by the TNF-α/TRADD pathway and the mechanism of pyroptosis induced by granzymes A and B. **B** Chemotherapy and CAR-T therapy induce cell death mediated by nonclassical pyroptotic pathways. **C** Interactions of PAMPs and DAMPs with pattern recognition receptors on the cell surface trigger the classical pyroptotic pathway, leading to the release of HMGB1, IL-1β, and IL-18. (Gzm A: Granzyme A; Gzm B: Granzyme B; PRRs: pattern recognition receptors; PAMPs: pathogen-associated molecular patterns; DAMPs: damage-associated molecular patterns; GSDMD-CT: GSDMD-C-terminus; and GSDME-CT: GSDME-C-terminus.)

Pyroptosis, a proinflammatory RCD pathway used in diverse types of cells, is triggered by human caspase-1, -3, -4, -5 (mouse caspase-11), -6, -8, and -9 and activated by a number of inflammatory bodies, including NLRP3 (Fig. 2) [35–40]. The crucial pyroptosis mediators—members of the gasdermin (GSDM) superfamily—are proteolytically activated by these caspases, after which they perforate the plasma membrane [41, 42]. Most members of the GSDM family (A-E) consist of an N-terminal pore-forming domain (PFD) and a C-terminal repressor domain [43, 44]. When a host is stimulated by various stimuli, GSDMs are cleaved by inflammatory caspases at the site of the linker region and liberate the PFD from the repressor domain [44, 45]. Consequently, the N-terminal PFD oligomerizes and forms pores in the cell membrane, leading to cell swelling, chromatin degradation, and expulsion of proinflammatory components (Fig. 2C) [46–48].

GSDMD has been reported to be cleaved by caspase-1, -4, -5, or -11, which is a hallmark of the canonical pyroptosis pathway [41]. PAMPs and DAMPs are detected by pattern recognition receptors, which activate downstream signaling pathways; as a result, ASCs are recruited to establish NLRP3 inflammasomes, which activate pro-caspase-1. Subsequently, activated caspase-1 cleaves GSDMD to free the PFD of GSDMD [49]. In addition, gram-negative bacteria predominantly trigger noncanonical pyroptosis through a mechanism distinct from inflammasomes and caspase-1 by activating human caspase-4/-5 (mouse caspase-11) to cleave GSDMD (Fig. 2B) [47, 50, 51]. Furthermore, the release of granzyme B (Gzm B) from chimeric antigen receptor (CAR)-T cells and chemotherapeutic medicines activates caspase-3, which in turn initiates the caspase-3/GSDME-mediated pyroptotic pathway, resulting in widespread pyroptosis [38, 40, 52]. Moreover, it has been demonstrated that Gzm B directly cleaves GSDME to cause pyroptosis, which subsequently activates the immune system to protect against tumors and slow tumor growth (Fig. 2A) [53]. Researchers have also discovered that natural killer (NK) cells and cytotoxic T lymphocytes kill cells expressing GSDMB via pyroptosis. Cleavage of GSDMB at the Lys229/Lys244 sites by granzyme A leads to lethal effects on target cells. GSDMB has been frequently detected in several tissues, including the epithelium of the digestive system [54]. Chen et al. even discovered that GSDMB binds directly to the caspase recruitment domain in caspase-4, promoting caspase-4 activity, which is necessary for the cleavage of GSDMD in noncanonical pyroptosis [55].

#### Ferroptosis

**Fig. 3:**
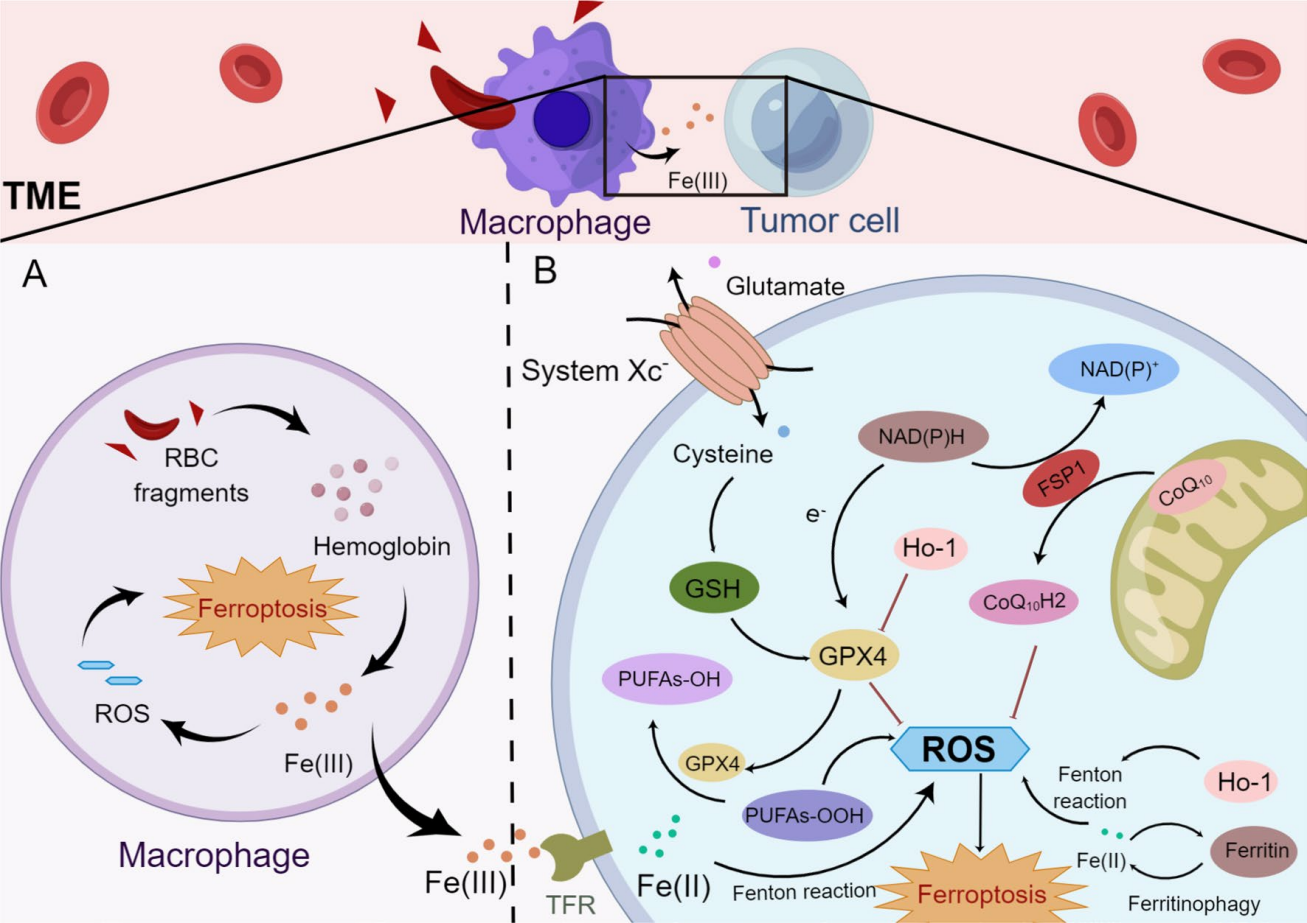
The interaction between MΦs and tumor cells in the TME and details of the ferroptosis pathway in tumor cells (by Figdraw). **A** MΦs engulf red blood cells and digest them into hemoglobin, which is further degraded into heme. Heme is catabolized into Fe(III) and Fe(III), which are released from MΦs or promote ROS production, leading to ferroptosis. **B** Ferroptotic cell death is induced by the inhibition of system Xc^−^, resulting in the abrogation of GSH biosynthesis and inactivation of GPX4, which subsequently cause cell death through excess lipid ROS production. PUFAs-OOH and Fe(II) facilitate tumor cell ferroptosis mediated by the Fenton reaction. (ROS: reactive oxygen species; system Xc^−^: cystine–glutamate antiporter; GSH: glutathione; GPX4: glutathione peroxidase 4; and RBC: red blood cell.)

Ferroptosis is a unique form of iron-dependent cell death that was originally discovered after tumor cells were exposed to a small-molecule chemical probe named erastin [56]. Morphological characteristics of ferroptosis involve reduced mitochondrial volume, fractured mitochondrial outer membrane, a decreased or absent mitochondrial crest, a normal-sized nucleus with no nuclear concentration, which distinguishes it from other modes of death [57]. Under normal conditions, lipoxygenases such as 12-/15-lipoxygenases often oxidize polyunsaturated fatty acids (PUFAs), but the lipid repair enzyme glutathione peroxidase 4 (GPX4) and its cofactor glutathione (GSH) cause a rapid decrease in the levels of lipoxygenase-oxidized PUFAs [58]. The ferroptosis process is induced by the suppression of the cystine–glutamate antiporter (system Xc^−^, comprising subunits SLC3A2 and SLC7A11), leading to decreased GSH biosynthesis and inactivation of GPX4 [59]. Subsequently, the cell dies due to overwhelming lipid peroxidation (Fig. 3B) [56, 57]. System X_C_^−^ inhibitors are categorized as class I ferroptosis-inducing substances, including sorafenib and sulfasalazine [60]. Class II ferroptosis-inducing substances are represented by RSL3, which covalently binds to and directly blocks GPX4, thereby rapidly inducing ferroptotic cell death [59].

Another ferroptosis defense system in cells was recently identified, the NAD(P)H-ferroptosis suppressor protein 1-ubiquinone (NAD(P)H-FSP1-CoQ_10_) pathway, which functions independently of the system X_C_^−^-GSH-GPX4 axis [61, 62]. FSP1, ferroptosis suppressor protein 1, is a flavoprotein that has been reported to induce apoptosis. CoQ_10_ is primarily synthesized in mitochondria, and in addition to its general importance in the mitochondrial electron transport chain, and its reduced form CoQ_10_H2 is a strong lipophilic antioxidant [63]. Hence, Kirill Bersuker and colleagues observed that FSP1 was recruited to the plasma membrane and then exerted an oxidoreductase function, reducing CoQ_10_. Subsequently, CoQ_10_H2 robustly halted the dissemination of lipid peroxides [62].

As the peroxidation of membrane phospholipids possessing PUFAs leads to ferroptosis [60], enzymes mediating the incorporation of PUFAs into phospholipids are important for ferroptotic cell death. For example, acyl-CoA synthetase long-chain family member 4 (ACSL4) leads to the enrichment of long PUFAs in cell membranes and is essential for the execution of ferroptosis [64]. Ferroptosis is also activated by components of the autophagy machinery, such ATG3, ATG5, ATG4B, ATG7, ATG13, and BECN1 [65]. Additionally, knockout or knockdown of the main genes governing autophagy reduces the effects of erastin on ferroptosis because intracellular ferrous iron levels are reduced [66]. Furthermore, Huang et al. documented that ferritinophagy, a proteolytic process through which ferritin is delivered to autophagosomes by NCOA4 [67], generates reactive oxygen species (ROS) and causes ferroptosis [68]. Cancer cells show high sensitivity to ferroptosis, which suggests a unique potential for cancer treatment. In fact, various primary cancers, such as liver cancer, clear cell renal cell carcinoma (RCC), and certain cancer cells with acquired drug resistance exert antitumor effects by inducing ferroptosis [69–71].

#### Cuproptosis

**Fig. 4:**
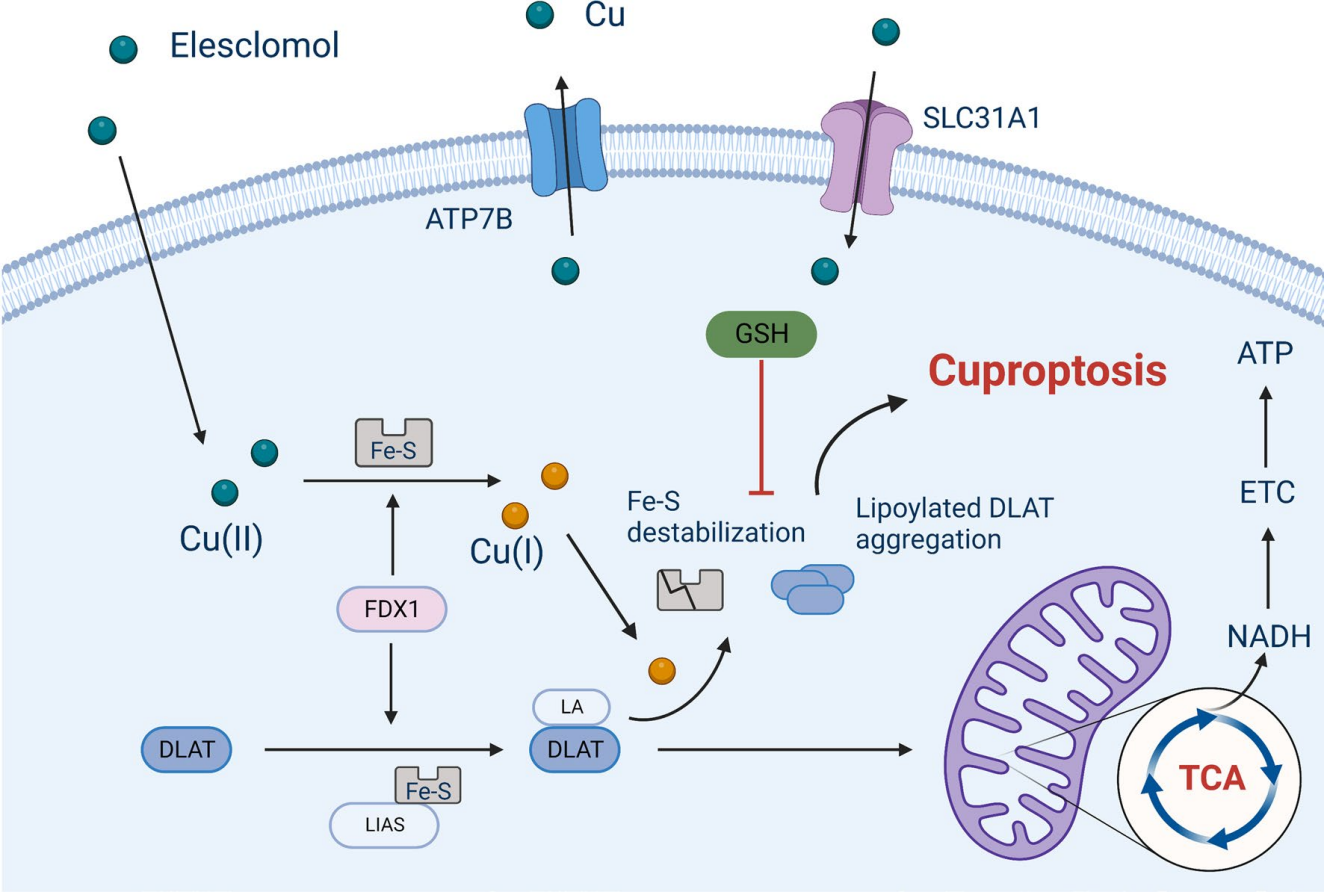
An excess Cu(II) supply can lead to cell pyroptosis (By BioRender). The uptake of Cu(II) into cells triggers pyroptosis via protein lipoylation, which is an important mechanism for the enzymatic function of proteins in the TCA (tricarboxylic acid) cycle

Recently, a novel cell death pathway triggered by copper (Cu), which differs from apoptosis, necroptosis, pyroptosis and ferroptosis, was discovered and coined “cuproptosis” by Peter Tsvetkov and colleagues in 2022 [25]. Cu is a crucial component for various physiological processes, especially tumor growth and metastasis, which have a heightened requirement for Cu [72]. Tsvetkov et al. described Cu-dependent death in 2019 while exploring the anticancer mechanism of elesclomol (a Cu ionophore) [73]. They found that the treatment of a multiple myeloma mouse model with elesclomol reduced the capability of the cancer cells to resist toxicity induced by proteasome inhibitors. Mechanistically, elesclomol-bound Cu(II) interacts with the mitochondrial enzyme ferredoxin 1 (FDX1) and is reduced to produce Cu(I), leading to increased levels of ROS [73, 74]. The lethality of elesclomol was first believed to be caused by lipid peroxidation [75]. Three years later, Tsvetkov and colleagues termed the unique form of Cu-dependent cell death cuproptosis, further supplementing the cell death mechanism induced by elesclomol [25]. The excess Cu(II) within cells can be transported to the mitochondria by ionophores, the FDX1 reduces Cu(II) to Cu(I). Increased amount of Cu(I) directly binds to lipoylated components (like DLAT) of the tricarboxylic acid (TCA) cycle, resulting in the lipoylated proteins aggregation and destabilization of Fe–S cluster proteins, leading to proteotoxic stress and, eventually, cell death (Fig. 4) [25]. Notably, the cell death pathway induced by Cu ionophores was not prevented by treatment with inhibitors of other already known cell death pathways, such as pan-caspase inhibitors (antiapoptotic compounds), ferrostatin-1 (an antiferroptotic compound), necrostatin-1 (an antinecroptotic compound), or N-acetyl cysteine (a suppressor of oxidative stress), suggesting that the cuproptosis mechanism differs from that of previously identified cell death pathways [25].

#### Cross talk among components of necroptosis, pyroptosis, ferroptosis, and cuproptosis

Accumulating evidence of widespread cross talk between key initiators, effectors and executioners of necroptosis, pyroptosis, ferroptosis, and cuproptosis has been reported. According to recent research, necroptosis activates the NLRP3 inflammasome by releasing potassium from the MLKL pore in macrophages (MΦs) [76]. The ZBP1 protein senses viral/endogenous nucleic acid ligands and triggers innate immune responses [77]. In detail, once ZBP1 is activated, RIPK3 and caspase-8 are recruited to activate the NLRP3 inflammasome, which initiates both necroptosis and pyroptosis [78–80]. In addition, the bioinformatics analysis reported by Miao et al. also indicated that the ZBP1, both of the cuproptosis-related and necroptosis-related gene signature, functions as a risk score for prediction of the low-grade glioma patients prognosis [81]. New outcomes from the research of Wei Gao and colleagues also revealed that the administration of elesclomol to CRC cells increased Cu(II) levels in mitochondria and downregulated the expression of the Cu(II) transporter ATP7A, leading to ROS accumulation. The procedure stimulated SLC7A11 degradation, which increased oxidative stress and led to ferroptotic death in CRC cells [75]. The recently constructed glucose oxidase (GOx)-engineered nonporous Cu(I) 1,2,4-triazolate ([Cu(tz)]) coordination polymer nanodrug GOx@[Cu(tz)] efficiently combined cancer starvation and cuproptosis induction [82]. The catalytic function of GOx@[Cu(tz)] can only be “turned on” and depletes glucose upon exposure to high GSH levels in cancer cells. The redox reaction between the released Cu(II) and intracellular GSH will induce GSH depletion and reduce Cu(II) to the Fenton agent Cu(I), which then catalyzes H_2_O_2_ to generate •OH via the Fenton reaction [83]. Subsequently, the exhaustion of glucose accompanied by GSH elimination further makes cancer cells more susceptible to cuproptosis [82] and probably ferroptosis.

### Diverse cell death modes in cancer biology: cell proliferation and metastasis

#### Brief overview of cancer cell proliferation and metastasis

Cell death is a physiological regulator of cell proliferation, and both processes exert profound effects on growth and development throughout life [48]. Cancer is characterized by the faulty regulation of cell division and death, which promotes uncontrolled tumor growth and replicative immortality [84]. An elevated cell proliferation rate and cell cycle abnormalities have been reported to be caused by inactivation of tumor suppressor genes such as CDKN2A, PTEN and TP53 [85, 86]. Because the induction of apoptosis alone via traditional anticancer therapeutic strategies does not completely eradicate cancer [84], studying methods to effectively inhibit abnormal cell proliferation and cell growth is important for cancer therapy.

Cancer metastasis is defined as the spread of tumor cells from their original location through the lymphatic system, blood vessels, or body cavities to colonize distant sites, establishing a local surviving cancer cell milieu and continual growth of secondary tumors [87]. Hanahan and Weinberg specified that one hallmark of cancer is “activated invasion and metastasis” [20]. The invasiveness of cancer cells into local tissue and seeding in areas distant from the original tumor to generate metastases are fundamental aspects of cancer malignancy [88], and metastasis remains the leading cause of mortality results from cancer [89]. In addition to cancer cell spread, metastasis facilitates cancer development via the degradation of the extracellular matrix, mediation of the epithelial-to-mesenchymal transition (EMT), promotion of tumor angiogenesis, and other processes [90, 91]. Numerous investigations have revealed that various RCD types are suppressed during tumor metastasis [92], and cell death caused by treatments administered locally or systemically effectively suppresses tumor metastasis [16]. Another widely held belief is that metastasis results from the spread of a malignant lesion through the activation of cellular reprogramming by microenvironmental stressors that promotes cell invasion and migration [93, 94]. Thus, investigating strategies to effectively suppress aberrant cell proliferation and cancer metastasis is essential to cancer treatment.

#### Necroptosis in proliferation and metastasis

Reports on the relationship between necroptosis and cancer have produced contradictory results, suggesting that necroptosis exerts different effects at different stages of cancer cell proliferation and metastasis. In cancer cells, the expression of major necroptotic pathway regulators is often downregulated, which has been found to be correlated with bad outcomes [95–99]. For example, according to Hockendorf et al., leukemogenesis is significantly accelerated after RIPK3 is knocked out in mice transplanted with bone marrow cells carrying a mutant AML driver gene, and the average lifespan of RIPK3-knockout mice is shorter than that of wild-type mice [100]. Additionally, low RIPK3 expression in colorectal cancer (CRC) patients and the reduced expression of MLKL in pancreatic adenocarcinoma and primary ovarian cancer have been reported to be correlated with reduced DFS and OS [97, 101, 102].

Clinical breast cancer tissues showed noticeably elevated the expression of TNFα, RIPK1, RIPK3, and MLKL at the mRNA and protein levels compared with their paired noncancerous tissues. Moreover, the pharmacological inhibition of necroptosis accelerates the proliferation and metastasis of breast cancer cells [103]. Han et al. discovered that the administration of compounds such as resibufogenin effectively suppresses the occurrence and metastasis of CRC by inducing RIPK3-mediated necroptosis [104]. Additionally, conventional anticancer therapies, such as platinum-based chemotherapy (cisplatin) and proteasome inhibitors (bortezomib), induce tumor cell necroptosis [105]. Furthermore, under certain conditions, necroptosis of cancer cells inhibits metastasis by triggering immunological responses against the tumor through DAMP generation [106].

However, in normal intestinal epithelial cells, the occurrence of MLKL-induced necroptosis disturbs gut homeostasis and results in inflammation [107]. Another study showed that melanoma cell-induced endothelial cell necroptosis significantly promotes the invasion and metastasis of malignant cells. Treating mice with the RIPK1 inhibitor necrostatin-1 or endothelial cell-specific deletion of RIPK3 significantly inhibits endothelial necroptosis and limits the extravasation and metastasis of malignant cells [108]. Similarly, Wang et al. reported that RIPK1 expression is significantly increased in both human lung cancer samples and mouse lung tumor models, suggesting that RIPK1 may exhibit an oncogenic function [109]. According to Liu et al., a higher level of MLKL phosphorylation is correlated with a poorer prognosis and shorter OS of patients with CRC and esophageal cancer [110]. Therefore, necroptosis of tumor cells exerts different effects on cell proliferation and spread and is not always beneficial in the treatment of cancer. In fact, many of the therapies that inhibit necroptosis have also shown good efficacy in the treatment of cancer, which will be introduced in detail at the end of this article.

#### Pyroptosis in proliferation and metastasis

Pyroptosis has significant therapeutic implications for several malignancies due to its profound effects on the invasion, proliferation, and metastasis of tumor cells. Based on several published investigations, pyroptosis-related modulators show tumor-suppressive activity against CRC [111], liver cancer [49], lung adenocarcinoma [112], and bladder cancer [113]. FL118, a camptothecin analog, inhibits the proliferation, invasion and metastasis of SW480 and HT129 cells by inducing caspase-1-dependent pyroptosis [114]. Additionally, lncRNAs are correlated with the regulation of pyroptosis. LncRNA RP1-85F18.6 is involved in promoting proliferation and invasion and suppressing pyroptosis in CRC cells, and knockdown of RP1-85F18.6 results in GSDMD cleavage to trigger pyroptosis [115]. Moreover, another study revealed that lower GSDMD expression is associated with a poorer CRC prognosis; furthermore, increased GSDMD expression effectively induces cell death [116]. The activation of pyroptosis by upregulating IFN-γ in mouse cancer cells inhibits tumor cell proliferation and enhances antitumor immunity in a mouse colon carcinoma cell line [54].

In recent years, many findings have been reported that pyroptosis suppresses the metastasis of cancer cells. Simvastatin is a statin with anticancer properties that has been implemented in the treatment of non-small cell lung cancer (NSCLC) [117]. The activated NLRP3 inflammasome and caspase-1 by simvastatin induces pyroptosis via the canonical pathway, inhibiting NSCLC cell migration [118]. Analogously, trichosanthin is a single-chain ribosome-inactivating protein extract from the root tuber of a Chinese medicinal herb [119] that inhibits cell growth and metastasis by promoting pyroptosis in NSCLC cells [120]. In addition, berberine is an isoquinoline quaternary alkaloid derived from medicinal plants that causes pyroptosis in HepG2 cells by inducing the expression of caspase-1 and preventing the migration and proliferation of HepG2 cells [121].

However, pyroptosis does not exert an absolutely positive therapeutic anticancer effect. A study revealed that elevated expression of GSDMC is also strongly associated with a worse prognosis for invasive breast carcinoma patients and exhibits a correlation with immune cell infiltration in the tumor [122]. Jianwei Gao and colleagues also found that increased GSDMD expression levels may increase the tumor size, promote more advanced tumor-node-metastasis stages, and affect survival rates. Moreover, GSDMD knockdown significantly restrains NSCLC cell proliferation via intrinsic mitochondrial apoptotic pathways and inhibits EGFR/Akt signaling [123]. Furthermore, as mentioned above, chemotherapy induces pyroptosis in cells which express high level of GSDME, while cells with low or no GSDME expression undergo apoptosis. However, in some tumor cell lines, the expression level of GSDME was lower than that in normal cell lines, leading to the accidental injury of normal tissues during chemotherapy [38, 124]. One explanation for the contradictory effects of pyroptosis is that whereas acute activation of pyroptosis results in necrotic cell death and inhibits tumor formation, persistent stimulation of pyroptosis promotes tumor progression [49]. In another study, abnormally upregulated GSDMB was reported to be critical for promoting the proliferation and invasiveness of bladder cancer cells [125]. Hergueta-Redondo et al. also discovered that a high level of GSDMB in breast cancer patients was related to tumor progression and a low treatment response rate [126]. Elucidating the precise mechanism by which pyroptosis reduces cancer growth and cancer cell proliferation is very important for the development of more-effective anticancer drugs.

#### Ferroptosis in proliferation and metastasis

Since a common concept related to the development of cancer is based on the specific mutations of oncogenes associated with the redox system [127], cancer cells show a higher level of Fe accumulation, which makes them more susceptible to the modulation of ferroptotic cell death than normal cells. Ferroptosis is a significant force modulating the growth and proliferation of certain types of tumor cells, such as diffuse large B cell lymphoma, RCC, melanoma, and ovarian cancer cells [59, 128, 129]. In 2015, Jiang et al. discovered that cancer cell ferroptotic death is induced by the p53 pathway when ROS are present at high or otherwise ectopic levels. P53 is significant in controlling cell proliferation. Mechanistically, activation of p53 substantially reduces cystine absorption by system X_C_^−^, which in turn inhibits intracellular GSH production, hence modulating the proliferation of tumor cells [130, 131]. Additionally, ART is a derivative of artemisinin that inhibits the proliferation of ovarian cancer cells by increasing the generation of ROS and triggering ferroptosis [132]. Furthermore, excessive proliferation of tumor cells is always accompanied by high ROS production, but these cells optimize ROS-driven proliferation via their effective antioxidant activity, enabling them to adapt and thrive under highly oxidative conditions while preventing ROS from reaching that threshold level that triggers ferroptosis [133, 134]. Analogously, tumor cells are also desensitized to ferroptosis by exporting iron through the secretion of ferritin-containing exosomes [135, 136]. Although ferroptosis significantly restricts tumor cell proliferation, effective compounds that induce ferroptosis must be identified while considering the adaptability of tumor cells.

Ferroptosis is also important in inhibiting cancer metastasis. The overexpression of the lncRNA BDNF-AS enhances peritoneal metastasis of gastric cancer by preventing ferroptosis [137]. Another study by Guan et al. also indicated that ferritinophagy-mediated ferroptosis and the KEAP1/NRF2/HO-1 pathway robustly contribute to EMT inhibition in gastric cancer cell lines [138]. Intriguingly, recent findings reported by Ubellacker and colleagues revealed that melanoma cells tend to spreading through the lymphatic system rather than through the bloodstream because lymph fluid contains higher expression levels of GSH and oleic acid and lower levels of free iron than blood plasma. This compositional difference contributes to decreased oxidative stress in lymph and inhibits the ferroptotic death of melanoma cells [139]. Additionally, Li et al. found the main feature of metastatic cells is sustained expression of GPX4, and GPX4 knockdown effectively induces ferroptosis and attenuates the enhanced tumorigenic and metastatic activity of malignant cells [92]. We assume that the induction of ferroptosis by inhibiting GPX4 and GSH might have superb potential for cancer therapy.

#### Cuproptosis in proliferation and metastasis

Cuproptosis, a Cu-induced cell death pathway, is highly associated with the mitochondrial metabolism [140] and plays a critical role in tumor cell proliferation, metastasis, and drug resistance [141, 142]. The levels of Cu accumulating in both serum and tumor tissues have been discovered to be markedly altered in individuals suffering from various malignancies, such as breast cancer [143], pancreatic cancer [144], thyroid cancer [145], leukemia [146], CRC [147], lung cancer [148], prostate cancer [149], and oral cancer [150]. These changes in Cu homeostasis may enhance tumor development or invasiveness or may confer resistance to treatment [151]. Recent investigations demonstrated that Cu is closely correlated with the expression level of hypoxia-inducible factor 1α [152, 153], which stimulates angiogenesis, and neovascularization in turn induces the production of vascular endothelial growth factor [154]. MEMO1, an oncogenic protein, was identified as an intracellular Cu-dependent protein that is required for breast cancer cell migration and invasion in vitro and spontaneous lung metastasis in vivo [155]. MEMO1 was presumed to bind to Cu(II) and promote ROS production through redox cycling, but Zhang et al. have shown that it preferentially binds to Cu(I) and shields cells from redox activity [155, 156]. Therefore, the identification of an appropriate method to block the binding site of Cu(I) on the MEMO1 protein might be a potential approach for releasing Cu ions and inhibiting the metastasis of tumor cells.

Cuproptosis may function by suppressing cancer cell proliferation and inhibiting metastatic events. For instance, in patients with oral squamous cell carcinoma who take betel nut, the related arecoline stimulation may inhibit cuproptosis, significantly increasing the viability of cancer-associated fibroblasts (CAFs) [157]. CAFs is crucial in cancer progression by contributing to the promotion of the EMT, cancer metastasis and chemotherapy resistance [158, 159]. Cuproptotic tumors also show decreased angiogenesis and are sensitive to sunitinib and sorafenib treatment [160]. To our surprise, cancer cells have evolved to activate a mechanism that prevents Cu-induced death to ensure their survival. Notably, Zhang et al. discovered that the key cuproptosis regulator FDX1 was profoundly downregulated in hepatocellular carcinoma (HCC) patients, leading to HCC cell resistance to cuproptosis [13]. In addition, lower expression of the FDX1 gene was reported to be closely associated with more advanced tumor-node-metastasis stages [161]. Furthermore, lower expression of FDX1 in various cancer types has been correlated with shorter survival times [13, 162, 163]. Despite these findings, cuproptotic events have not been well documented in diverse cancers. Therefore, additional in vitro and in vivo experiments are needed to confirm a role for cuproptosis in the proliferation and metastasis of cancer cells.

### Pleiotropic functions of cell death in the TME

Events in TME have been extensively correlated to tumor development, progression, and responses to chemotherapy and antiangiogenic therapy [164–166]. The TME includes noncancerous cells, including components that are also present in the tumor, such as immune cells, CAFs, endothelial cells, mesenchymal stroma/stem cells (MSCs), extracellular matrix compounds, and soluble products such as chemokines, cytokines, growth factors, and extracellular vesicles [167, 168]. According to experimental data, tumor immunity within the TME may be influenced by necroptosis, pyroptosis, ferroptosis, and cuproptosis [13, 169]. For example, the novel RCDs increased infiltration of tumor-infiltrating lymphocytes and antitumor immunity have been reported in the tumors of long-term small cell lung cancer survivors [170]. It is widely believed that the treatments with immune checkpoint inhibitors (ICIs) are not effective in “cold tumors,” which are characterized by the lack of T cell infiltration, while “hot tumors” with significant T cell infiltration contribute to better ICI efficacy [171]. In this section, we explore various types of cell death in the TME and show how the interaction of host immune cells with these cells affects tumor progression and cancer treatment.

#### Cancer cell death influences immune cell infiltration into the TME

##### Tumor necroptosis influences the TME

The induction of necroptosis in tumor cells has been shown to contribute to an autophagy-mediated increase in DAMPs, which subsequently triggers immunosurveillance [172]. Notably, the necrotic cells transplanted into the TME stimulate the antitumor immune response mediated by BATF3 + cDC1- and CD8 + leukocytes and are accompanied by tumor-associated antigen-presenting cells, which increase the tumor antigen load (Fig. 4) [173]. The enhanced immunogenicity and vaccine efficacy obtained after inducing tumor necroptosis may constitute an approach to the development of cancer vaccines. Furthermore, Aaes et al. documented that vaccinating an experimental mouse model with necroptotic cancer cells induced potent antitumor immunity by promoting the maturation of dendritic cells (DCs), inducing cross-priming of cytotoxic T cells and IFN-γ production in response to tumor antigen stimulation [174]. Moreover, derepression of TRIM28 activity by RIPK3 activation in malignant cells leads to increased production of immunostimulatory cytokines within the TME, contributing to robust cytotoxic antitumor immunity [175]. Hence, de novo necroptotic death creates an inflammatory milieu that modifies tumors that are responsive to ICIs [176].

**Fig. 5:**
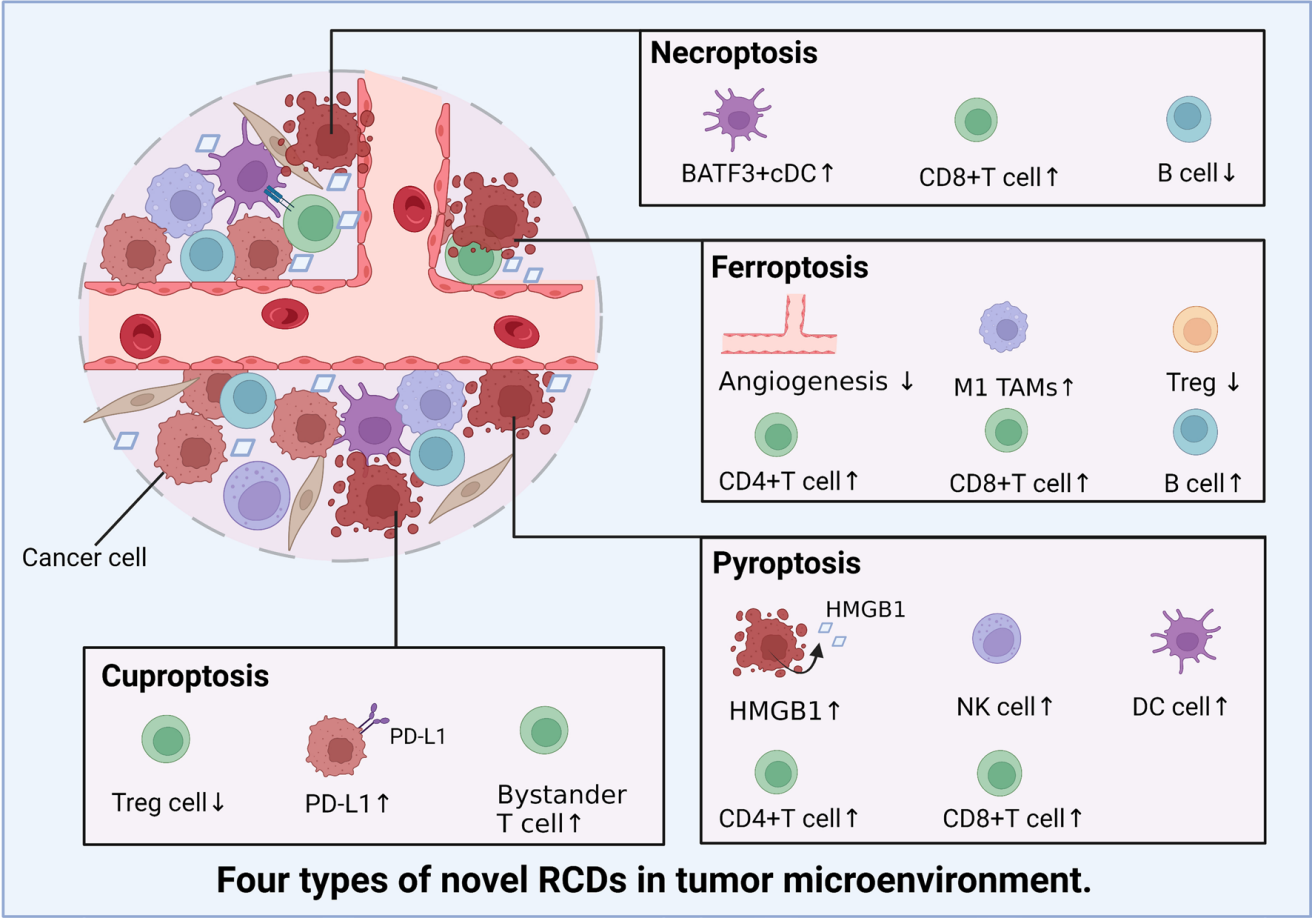
Summary of novel RCD modalities in tumor cells that influence the TME (By BioRender). We summarize the effects of tumor cell necroptosis, pyroptosis, ferroptosis, and cuproptosis on the number of immune cells and the levels of immune-related factors in the TME

He et al. discovered 8 differentially expressed necroptosis-related genes in tumors compared with their expression in normal tissues, thereby revealing a prognostic signature they called the NRS score. NRS scores were positively correlated with the number of follicular helper T cells, CD8 + T cells, resting mast cells, M1 MΦs, and M2 MΦs. M1 MΦs typically exert antitumor effects, but M2 MΦs are proposed to be protumorigenic. He et al. found necroptosis might also shield tumors from antitumor immune responses by fostering an immunosuppressive milieu and immune escape mechanisms [177, 178]. Analogously, in pancreatic ductal adenocarcinoma, RIPK3 expression is significantly upregulated compared with that in normal tissues, whereas RIPK3 deletion mitigates the expression of the chemokine CXCL1 in vivo and in vitro. Furthermore, RIPK3 deletion diminishes the infiltration of immunosuppressive myeloid cell subsets (tumor-associated MΦs (TAMs), myeloid-derived suppressor cells (MDSCs) and DCs), and the proportions of T cells and B cells are increased (Fig. 5) [179]. The increases in percentage of T cell and B cell number indicate potent anticancer effects [180].

Therefore, the effect of necroptosis on the TME cannot be fully determined, and more evidence obtained from a combination of basic experiments and clinical trials is still required to identify the specific function of necroptosis.

##### Tumor pyroptosis affects the TME

As mentioned above, compared with necroptosis and ferroptosis, pyroptosis is a more common mechanism of immune defense [181]. Pyroptosis is intimately related to immune cell infiltration into the TME in various cancers. For instance, combinations of BRAF inhibitors and MEK inhibitors (BRAFi + MEKi) modulate the TME by triggering pyroptosis [182]. BRAFi + MEKi have been approved by the FDA to treat BRAF V600E/K-mutant melanoma, but this treatment leads to a certain degree of resistance [183]. Erkes and colleagues revealed that BRAFi + MEKi therapy enhances GSDME cleavage and HMGB1 release. HMGB1 sufficiently induces the infiltration of DCs to expand the proportions of CD4 + and CD8 + T cells, particularly activated (CD44 +) and proliferating (Ki-67 +) T cells, which exert antitumor effects (Fig. 5). Intriguingly, in a BRAFi + MEKi-resistant disease context, intratumoral T cell infiltration is decreased, which is reversed by pyroptosis-inducing chemotherapy [182]. Another study further characterized a distinct prognostic factor for lung adenocarcinoma based on PRGs called the Pyro-score, showing that a low Pyro-score indicates increased immune cell infiltration. And patients with an increased number of infiltrating immune cells exhibit higher sensitivities to anti-PD-1/L1 immunotherapy [184]. Zhang et al. discovered that pyroptosis mediated by GSDME cleavage suppresses tumors by increasing the numbers of tumor-infiltrating NK cells and CD8 + T lymphocytes, as well as by inducing phagocytosis by TAMs (Fig. 5) [53]. However, cancer cells have evolved two strategies to prevent the tumor-suppressing effect of GSDME: (1) epigenetically suppressing GSDME expression [185] and (2) loss-of-function mutations [186]. Indeed, Cai et al. found that the natural product triptolide potently eliminates head and neck cancer cells by inducing GSDME-mediated pyroptosis, which is significantly attenuated after GSDME gene silencing [187]. Therefore, solutions for overcoming these problems will help scientists leverage pyroptosis to improve cancer treatment. Recent findings from a study by Fan and colleagues suggest that harnessing decitabine to demethylate the GSDME gene in malignant cells before inducing pyroptosis effectively overcomes the epigenetic restriction of GSDME [188]. Additionally, strategies have been developed to package recombinant adeno-associated viruses expressing the PFD of GSDM to induce pyroptosis and prolong the life span of cancer models [189].

In spite of the positive experimental findings suggesting the antitumor role of pyroptosis in TME, the study from Tan et al. demonstrated that HMGB1 released from pyroptotic cell death contributes to the tumorigenesis of colitis-associated colorectal cancer through activating ERK1/2 pathway [190]. Activation of ERK1/2 signaling plays a protumorigenic role in modulation of the TME via inducing M2 MΦs polarization [191]. Therefore, it is important to consider the possibility of failure as we continue to research anticancer properties of pyroptosis.

##### Tumor ferroptosis alters the TME

Ferroptotic cancer cells generate some “find-me” and “eat-me” immunostimulating signals, particularly DAMPs, which robustly recruit DCs, MΦs, and other immune cells properly to the site of dying tumor cells [192, 193]. Another recent study also revealed that ferroptosis induced by a kind of nanomodulators attenuated the self-renewal capability of cancer and downregulated the expression of genes related to angiogenesis (Fig. 5) [194]. Therefore, ferroptosis shows anticancer effect via promoting immunogenicity and inhibiting metastasis-related genomic expression. It was also discovered that early ferroptotic cancer cells can accelerate the phenotypic development of DCs and elicit a vaccination-like response [195]. Additionally, according to research by Géraldine Luis and colleagues, fatty acid-binding protein-4 in TME which increases the production of lipid droplets in cancer cells, and stearoyl-CoA desaturase-1 (SCD1) expressed by cancer cells, cooperatively protects cancer cells from oxidative stress-induced ferroptosis and promotes tumor recurrence [196]. Furthermore, some chemotherapeutic medications, such as cisplatin, targeted medications, such as sorafenib, and radiotherapy strongly induce ferroptosis [197–199], which encourages the infiltration of immune cell and raises the immunogenicity of immune-desert tumors, ultimately improving the efficacy of ICI immunotherapy. Tumor cells are significant in constructing immune-stimulating microenvironment, which opens the door for the emergence of innovative solutions to cancer immunotherapy.

Bioinformatics study revealed 7 differentially expressed FRGs in papillary thyroid carcinoma that are positively correlated with a TME enriched with infiltrating immune cells. The three most highly activated subtypes of cells were B cells, CD8 + T cells, and CD4 + T cells (Fig. 5) [200]. Moreover, another risk signature score based on FRGs revealed that lymph node invasion and venous invasion events were more common in high-scoring ovarian cancer samples. Furthermore, infiltrated immune cells and stromal cells were more frequent in the high-scoring group [201]. Xu et al. selected 9 differentially expressed long noncoding RNAs associated with ferroptosis to develop a prognostic signature for patients with HCC, and an increase in the expression of the immunological checkpoint protein B7H3 was identified in the high-risk group [202].

However, cancer cells that undergo ferroptotic death are also associated with the release of PGE2, a significant immunosuppressant that disrupts the anticancer activities of NK cells, DCs, and cytotoxic T cells [59, 203]. Demuynck et al. proposed that cancer cell ferroptosis may substantially increase the levels of oxidized lipids, leading to decreased phagocytosis and antigen cross-presentation by DCs and thus potentially favoring tumor cell evasion of immune surveillance [204]. Additionally, NRF2—nuclear factor erythroid 2-related factor 2, has been proved to inhibit ferroptotic cell death, which can be stimulated by oncoproteins including c-Myc, K-RAS, and B-raf. Intriguingly, downregulating NRF2-targeted genes could increase ferroptosis in the TME and promote cancer progression [205, 206]. Because of persistent uncertainty, we must continue to view the role of tumor cell ferroptosis in the tumor immune microenvironment with skepticism.

##### Tumor cuproptosis affects the TME

Cuproptosis is associated with immune cell infiltration. As shown in a recent study, melanoma patients with higher expression of CRGs experienced a longer OS. One prominent CRG is LIPT1, whose expression is positively linked to PD-L1 expression and negatively correlated with regulatory T cell infiltration (Fig. 5) [207]. Increased PD-L1 expression suggests that the combination of cuproptosis induction via immune checkpoint blockers may show better efficacy. Additionally, in esophageal carcinoma, higher expression of CRGs was anomalously correlated with an increased number of infiltrating bystander T cells (Fig. 5) [208]. According to Zhang et al., lower CRG expression in HCC patients correlates with an increase in protumor immune components in tumors; however, no change in the percentage of antitumor immune cells was reported [13]. In addition to the role of CRGs in the establishment of TME with antitumor effects, other studies have also shown that cuproptosis-related lncRNAs are also related to the changes of immune cell infiltration. Wang et al. collected a total of 16 cuproptosis-related lncRNAs and constructed high- and low-risk prognostic signatures based on the nomogram and heatmap of cuproptosis-related lncRNAs. They found the high-risk patients have a greater potential for immune escape and less response to cancer immunotherapy of lung adenocarcinoma [209]. Currently, cuproptosis is presumed to play a certain role in shaping an antitumor immune environment, but whether Cu-dependent death exerts an inhibitory effect on cancer immunotherapy remains to be determined. Therefore, clarifying the function of cuproptosis is crucial for the formulation of future combination therapies.

#### Cell fates in the TME

A 2016 study revealed that alanine released from stroma-associated pancreatic stellate cells by autophagy was a substitute carbon source that fueled the TCA cycle in pancreatic ductal adenocarcinoma. This change in fuel source reduced the tumor cell reliance on glucose and nutrients obtained from serum, which are limited in the pancreatic TME [210]. Based on this finding, we logically suspect that a novel RCD pathway of noncancer cells in the TME may affect cancer cell survival. Therefore, we investigated this hypothesis in detail.

Interestingly, novel forms of RCD for cells that compose the TME profoundly influence the tumor fate. For instance, RIPK3 downregulation in TAMs induces fatty acid oxidation and M2 MΦ polarization in the TME, facilitating HCC tumorigenesis [211]. In addition, Huanrong Lan and colleagues revealed that oxaliplatin resistance in CRC results from the necroptotic evasion of M2 MΦs. Mechanistically, the expression of the methyltransferase METTL3 is increased in oxaliplatin-resistant CRC tissues, and METTL3-mediated N^6^-adenosine methylation significantly inhibits TRAF5-induced necroptosis both in vitro and in vivo [212]. Thus, the necroptosis of MΦs tends to exert a positive antitumorigenic effect.

The discovery that the serine protease inhibitor Val-boroPro (also called talabostat or PT-100) cleaves the substrate at proline has generated significant interest in this compound as a potential anticancer drug. Val-boroPro achieves its anticancer effects by activating pro-caspase-1, which is subsequently cleaved to activate GSDMD and induces the pyroptosis of monocytes and MΦs [213].

As shown in the study by Hage et al., sorafenib induces pyroptosis in MΦs to stimulate HCC cell killing [214]. Specifically, sorafenib robustly increases the activity of caspase-1, activating GSDM and inducing MΦ pyroptosis. Subsequently, NK cells are activated when cocultured with sorafenib-treated MΦs, and the interplay of MΦs and NK cells induces HCC cell death. Moreover, various cytokines are released from pyroptotic immune cells, including IL-18, which shows established anticancer activity by enhancing the type 1 immune response and can thus be utilized in cancer immunotherapy [215, 216].

MΦs engulf red blood cells and digest them to generate hemoglobin, which is further degraded into heme. Heme is catabolized into iron, which either promotes ROS generation or lipid peroxidation. Through ferroportin, the iron produced by heme is discharged into the environment, increasing the iron level in the TME (Fig. 3A) [217]. Then, iron promotes the Fenton reaction and generates hydroxyl radicals, which cause tumor cells to undergo ferroptosis [218]. Therefore, macrophages increase the content of iron in the TME through their own ferroptosis and promote the subsequent ferroptosis of tumor cells, thus showing a powerful antitumor effect. Ferroptosis of MDSCs was demonstrated to be crucial in fighting malignancies, but Zhu et al. found that N-acyl-sphingosine amidohydrolase (ASAH2) is expressed at high levels in MDSCs in colon carcinoma. ASAH2 reduces MDSC ferroptosis by reducing p53 stability, upregulating Hmox1 expression, and inhibiting lipid ROS production in the TME. The ASAH2 inhibitor NC06 induces ferroptosis in MDSCs by inhibiting ceramidase activity. Animal models confirmed that NC06 inhibits the infiltration of MDSCs into transplanted tumors by promoting MDSC ferroptosis and thus inhibits tumor growth [219]. Furthermore, ferroptosis mediated by tumor-infiltrating lymphocytes significantly enhances the efficacy of ICIs [220, 221].

Since cuproptosis is a novel RCD, determining whether it occurs among noncancerous TME cells is a challenge. However, upon Cu stimulation, exosomes secreted by MΦs increase angiogenesis mediated by endothelial cells in vitro and in vivo [222]. Ryuhei Takemoto and colleagues also found that overexpression of lysyl oxidase, a Cu-containing enzyme, in human leukemic THP-1-cell-derived M2 MΦs promotes tumor metastasis [223]. Therefore, immune cell cuproptosis may have a multifaceted role in TME, and we are awaiting rational animal and cellular investigations to elucidate this role.

### Current and future therapeutics targeting different cell death pathways

As we previously discussed, tumor cells and other cells in the TME that undergo necroptosis, pyroptosis, ferroptosis, or cuproptosis possibly contribute to strong antitumor immunity. Additionally, mechanisms for bypassing the apoptosis signaling pathways that cause the death of cancer cells have attracted considerable attention for their use in anticancer therapy [224]. Therefore, we describe small-molecule compounds and other agents targeting novel mechanisms of cell death that might be employed in cancer therapy (Table 1), and we emphasize the therapeutic approaches that have been tested in clinical trials to date (Table 2).

#### Agents targeting novel cell death pathways

Targeting necroptosis, pyroptosis, and ferroptosis to develop new anticancer medications for therapeutic use has been a long process, and recently, compounds inducing cuproptosis have shown promise as anticancer strategies [225, 226]. In Table 1, we summarize 85 types of therapeutic agents that exert an effect on the mechanisms of newly discovered RCD modalities that have been tested in vivo and/or in vitro.

**Table 1:** Summary of agents targeting novel RCDs in cancer-related preclinical studies

Classification	Compound	Cell death pathways	Mechanisms	Cancer type	References
Small compound	Shikonin	Necroptosis induction	Increases ROS production and upregulates RIPK1, RIPK3, and MLKL	Nasopharyngeal carcinoma	[227]
FDA-approved drug	FTY720	Necroptosis induction	Targets I2PP2A/SET and activates PP2A/RIPK1	Lung cancer	[228]
NPs	Graphene oxide NPs	Necroptosis induction	Increases the activity of RIPK1, RIPK3, and HMGB1	Colon cancer	[229]
NPs	Selenium NPs	Necroptosis induction	Increases ROS and RIPK1 production	Prostate adenocarcinoma cells	[230]
Small compound	Emodin	Necroptosis induction	Enhances TNF/RIPK1/RIPK3 signaling	Glioma	[231]
Small compound	Ophiopogonin D′	Necroptosis induction	Upregulates RIPK1	Prostate cancer	[232]
Small compound	Resibufogenin	Necroptosis induction	Upregulates RIPK3	CRC	[104]
Small compound	Bufalin	Necroptosis induction	Upregulates RIPK1/RIPK3	Breast cancer	[233]
Small compound	Tanshinol A	Necroptosis induction	Increases intracellular ROS and MLKL levels	Lung cancer	[234]
FDA-approved drug	Chloroquine (CQ)	Necroptosis induction	Upregulates RIPK3	CRC	[235]
NPs	Hyaluronic acid -modified, lipid-coated PLGA NPs loaded with mRIP3-pDNA	Necroptosis induction	Upregulates RIPK3	CRC, breast cancer, melanoma	[235]
Small compound	CBL0137	Necroptosis induction	Activate ZBP1	Liver cancer, breast cancer, colorectal cancer, cervical cancer, melanoma	[236]
Small compound	Smac mimetics	Necroptosis induction	Antagonizes inhibitor of apoptosis proteins (cIAPs)	Burkitt’s lymphoma	[237]
NPs	Fe(III)-shikonin supramolecular nanomedicine (FSSN)	Necroptosis/ferroptosis induction	1. Decreases GSH levels 2. Promotes RIPK1-RIPK3 complex generation	Breast cancer	[238]
Small compound	Necrosulfonamide (NSA)	Necroptosis inhibition	1. Inhibits MLKL	Breast cancer	[110]
Small compound	PK68	Necroptosis inhibition	1. Inhibits RIPK1	Melanoma, lung carcinoma cells	[239]
Small compound	Necrostatin-1	Necroptosis inhibition	1. Inhibits RIPK1	Colitis-associated CRC	[240]
FDA-approved drug	Metformin	Pyroptosis induction	1. Induces GSDMD-mediated pyroptosis by targeting the miR-497-PELP1 axis 2. Causes mitochondrial dysfunction and activates the AMPK/sirtuin1/NF-κB pathway	Breast cancer, esophageal squamous cell carcinoma	[241, 242]
Small compound	Tetraarsenic hexoxide	Pyroptosis induction	Enhance the production of mitochondrial reactive oxygen species (ROS) by inhibiting phosphorylation STAT3	Breast cancer	[243]
FDA-approved drug	Docosahexaenoic acid	Pyroptosis induction	Increases caspase-1 levels and activates GSDMD	Breast cancer	[244]
Small compound	Polyphyllin VI	Pyroptosis induction	Increases ROS production	NSCLC	[245, 246]
FDA-approved drug	Simvastatin	Pyroptosis induction	Increases caspase-1 expression	NSCLC	[118]
Small compound	Galangin	Pyroptosis induction	Increases levels of the GSDME N-terminus	Glioblastoma multiforme	[247]
Investigational drug	Lobaplatin	Pyroptosis induction	Induces ROS production and JNK phosphorylation	Colon cancer	[248]
Investigational + FDA-approved drugs	Mirdametinib + Vemurafenib	Pyroptosis induction	Inhibits the MEK/ERK 1/2 pathway and promotes Bim/Bmf-mediated mitochondrial depolarization	Melanoma	[182]
Small compound	Miltirone	Pyroptosis induction	Inhibits the MEK/ERK 1/2 pathway and promotes ROS accumulation	Hepatocellular carcinoma	[249]
NPs	Biomimetic nanoparticles	Pyroptosis induction	Induces the mitochondrial damage and activates caspase-3	Breast cancer	[250]
NPs	DAC + LipoDDP	Pyroptosis induction	Activates the caspase-3 pathway	Breast cancer	[188]
NPs	Synthetic RIG-I agonist	Pyroptosis induction	Activates STAT1 and NF-κB	Breast cancer	[251]
NPs	CXCR4-targeted NPs	Pyroptosis induction	Upregulates caspase-11 and NLPR3 expression	Colorectal cancer	[252]
NPs	Lip MOF NPs	Pyroptosis induction	Cleaves GSDMD and increases IL-1β levels	Cervical cancer	[253]
NPs	Arsenic trioxide NPs	Pyroptosis induction	Increases caspase-3 expression	Hepatocellular carcinoma	[254]
NPs	ROS-responsive polyion complex	Pyroptosis induction	Induces oxidative DNA damage	Breast cancer	[255]
FDA-approved drug	Paclitaxel	Pyroptosis induction	Activates caspase-3/GSDME	Lung cancer	[256]
Small compound	Anthocyanin	Pyroptosis induction	Increases caspase-1 levels	Oral squamous cell carcinoma	[257]
Small compound	JQ-1	Pyroptosis induction	Activates the NF-κB-NLRP3-caspase-1 pathway	Renal cell carcinoma	[258]
Investigational drug	Talabostat	Pyroptosis induction	Activates caspase-1	Cervical cancer, fibrosarcoma, breast cancer	[213]
Small compound	Ivermectin	Pyroptosis induction	Activates P2X4/P2X7-gated pannexin-1 channel	Breast cancer	[259]
Small compound	Ophiopogonin B	Pyroptosis induction	Activates caspase-1/GSDMD pathway	Lung cancer	[260]
Small compound	GW4064	Pyroptosis induction	Activates caspase-3/GSDME pathway	CRC	[261]
FDA-approved drug	Doxorubicin	Ferroptosis/pyroptosis induction	Increases lipid ROS levels	HCC, CRC	[262, 263]
Investigational drug	Anti-GSDMB antibody	Pyroptosis inhibition	Inhibits GSDMB	Breast cancer	[264]
Small compound	Dimethyl fumarate	Pyroptosis inhibition	Inhibits GSDMD	Breast cancer	[265]
NPs	Zero-valent iron NPs	Ferroptosis induction	Converts zero-valent iron to Fe(II) and facilitates Fenton reactions	Oral cancer	[266]
NPs	Fe-CO@Mito-PNBE	Ferroptosis induction	(1) Enhances ROS generation (2) Releases Fe(III)/Fe(II) ions in the TME and triggers the Fenton reaction	Cervical cancer, breast cancer	[267]
Small compound	IMCA	Ferroptosis induction	Downregulates SLC7A11 expression and reduces cellular cysteine uptake	Colorectal cancer	[268]
Small compound	Imidazole ketone erastin	Ferroptosis induction	Inhibits system X_C_^−^	Diffuse large B cell lymphoma	[269]
Small compound	Hydrolysis product 4 of open chain epothilone analogs	Ferroptosis induction	Inhibits system X_C_^−^	Fibrosarcoma, rhabdomyosarcoma	[270]
Small compound	ML210	Ferroptosis induction	Suppresses GPX4	Melanoma, pancreatic cancer, lung adenocarcinoma, fibrosarcoma, renal cell carcinoma, pancreatic cancer	[271]
Small compound	ML162	Ferroptosis induction	Suppresses GPX4	Fibrosarcoma	[59]
Small compound	DMOCPTL	Ferroptosis induction	Suppresses GPX4	Breast cancer	[272]
Small compound	FIN56	Ferroptosis induction	Inhibits CoQ_10_ biosynthesis	Fibrosarcoma	[273]
Small compound	FINO_2_	Ferroptosis induction	Suppresses GPX4	Fibrosarcoma	[274]
Small compound	Albiziabioside A derivative	Ferroptosis induction	Suppresses GPX4	CRC	[275]
Small compound	Trigonelline	Ferroptosis induction	Decreases GSH levels	Head and neck cancer cells	[276]
FDA-approved drug	Glutamate	Ferroptosis induction	Inhibits system *X*_C_^−^	Fibrosarcoma	[56]
NPs	MFC-Gem	Ferroptosis induction	Decrease GSH levels	Pancreatic cancer	[277]
Small compound	Erianin	Ferroptosis induction	Activates Ca^2+^/CaM signaling	Lung cancer	[278]
FDA-approved drug	Etoposide	Ferroptosis induction	Decreases GPX4 levels	Myelogenous leukemia	[279]
NPs	FePt/MoS2 nanocomposites	Ferroptosis induction	Supplies Fe(II) and accelerates Fenton reactions	Cervical carcinoma, breast cancer	[280]
FDA-approved drug	Sorafenib	Ferroptosis induction	Inhibits cystine–glutamate antiporter (system *X*_C_^−^)	Fibrosarcoma, Ewing’s sarcoma, lung cancer, osteosarcoma	[281]
Investigational drug + FDA-approved drug	Siramesine + lapatinib	Ferroptosis induction	Inhibits ferroportin-1	Breast cancer	[282]
FDA-approved drug	Cisplatin	Ferroptosis induction	Depletes glutathione (GSH) and inactivates GPX4	NSCLC, colon cancer	[197]
FDA-approved drug	Decitabine	Ferroptosis induction	Decreases GSH levels and inhibits GPX4 activity	Myelodysplastic syndrome	[283]
Synthetic exosomes	Erastin@FA-exo	Ferroptosis induction	Suppresses GPX4 expression and upregulates cysteine dioxygenase	Breast cancer	[284]
FDA-approved drug	Apatinib	Ferroptosis induction	1. Inactivates GPX4 2. Upregulates ELOVL6/ACSL4 3. Inhibits the VEGFR2/Nrf2/Keap1 pathway	Gastric cancer, colorectal cancer, glioma	[285–287]
FDA-approved drug	Altretamine	Ferroptosis induction	Inactivates GPX4	Diffuse large B cell lymphoma	[288]
FDA-approved drug	Neratinib	Ferroptosis induction	Increases ferritin levels	Breast cancer	[289]
Investigational drug	APR-246	Ferroptosis induction	Decreases GSH biosynthesis	Acute myeloid leukemia	[290]
Investigational drug	Flubendazole	Ferroptosis induction	Inhibits SLC7A11 transcription	Prostate cancer	[291]
FDA-approved drug	Sulfasalazine	Ferroptosis induction	Inhibits system X_C_^−^	Head and neck cancer	[292]
FDA-approved drug	Statins	Ferroptosis induction	Inhibits CoQ_10_ biosynthesis	NSCLC	[293, 294]
FDA-approved drug	Dihydroartemisinin	Ferroptosis induction	Inhibits GPX4	Leukemia	[295, 296]
Small compound	RSL3	Ferroptosis induction	Suppresses GPX4	CRC	[297]
Small compound	MMRi62	Ferroptosis induction	Degrades mutant p53 and ferritin heavy chain	Pancreatic cancer	[298]
FDA-approved drug	Artesunate	Ferroptosis induction	Promotes ROS signaling and lipid peroxidation	Pancreatic cancer	[299]
Investigational drug	Buthionine sulfoximine	Ferroptosis/cuproptosis induction	Decreases GPX4 levels	Hepatocellular carcinoma, lung cancer	[140, 300]
Small compound	5-aminolevulinic acid	Ferroptosis induction	Inhibits GPX4 and increase heme oxygenase 1(HO-1)	Esophageal squamous cell carcinoma	[301]
Chinese patent medicine	Shuganning injection	Ferroptosis induction	Increases HO-1	Triple-negative breast cancer	[302]
Small compound	Trichothecin	Ferroptosis induction	Upregulates RIPK3	CRC, Burkitt lymphoma	[303]
NPs	ZVI-NP	Ferroptosis induction	Inactivates NRF2	Lung cancer	[194]
NPs	2,2,6,6, tetramethylpiperidine-*N*-oxyl capped TiO_2_ nanorods (TiO_2_ NRs) + radiotherapy	Ferroptosis inhibition	Induces GSH	Breast cancer	[304]
Small compound	Liproxstatin-1	Ferroptosis inhibition	Slow the accumulation of lipid hydroperoxides	Pancreatic cancer	[305]
Investigational drug	Elesclomol	Cuproptosis induction	1. Increases copper levels and targets lipoylated TCA cycle proteins 2. Targets copper-transporting ATPase 1 and regulates ferroptosis	NSCLC, lung adenocarcinoma, pancreatic cancer, papillary thyroid carcinoma, ovarian cancer, colorectal cancer	[25, 75]
NPs	CuET NPs	Cuproptosis induction	Reduces FDX1 expression	NSCLC	[306]

#### Agents inducing novel cell death pathways

##### Approved and investigational drugs inducing novel RCD pathways

According to recent investigations, many clinically approved medications exert potent antitumor effects by inducing (or inhibiting) inflammatory RCD modalities in preclinical studies [169]. CQ has been shown to upregulate endogenous RIPK3 in CRC cell lines, and Hou et al. reported that necroptosis mediates this process, which is not affected by apoptosis inhibitors [235]. Interestingly, shikonin, a naphthoquinone product synthesized from the roots of a Chinese medicinal herb, induces nasopharyngeal carcinoma cell necroptosis in a dose-dependent manner [227]. Mechanistically, shikonin increases ROS production and upregulates the expression levels of RIPK1, RIPK3, and MLKL, which prompts necroptosis in apoptosis-resistant tumor cells [307]. However, the activation of necroptosis can also be mediated by the modulation of the upstream signaling pathways. For instance, the sphingosine analog FTY720, also called fingolimod, induces necroptosis in human lung cancer cells by binding to inhibitor 2 of PP2A (I2PP2A/SET oncoprotein), thus activating the PP2A/RIPK1 pathway [228].

**Fig. 6:**
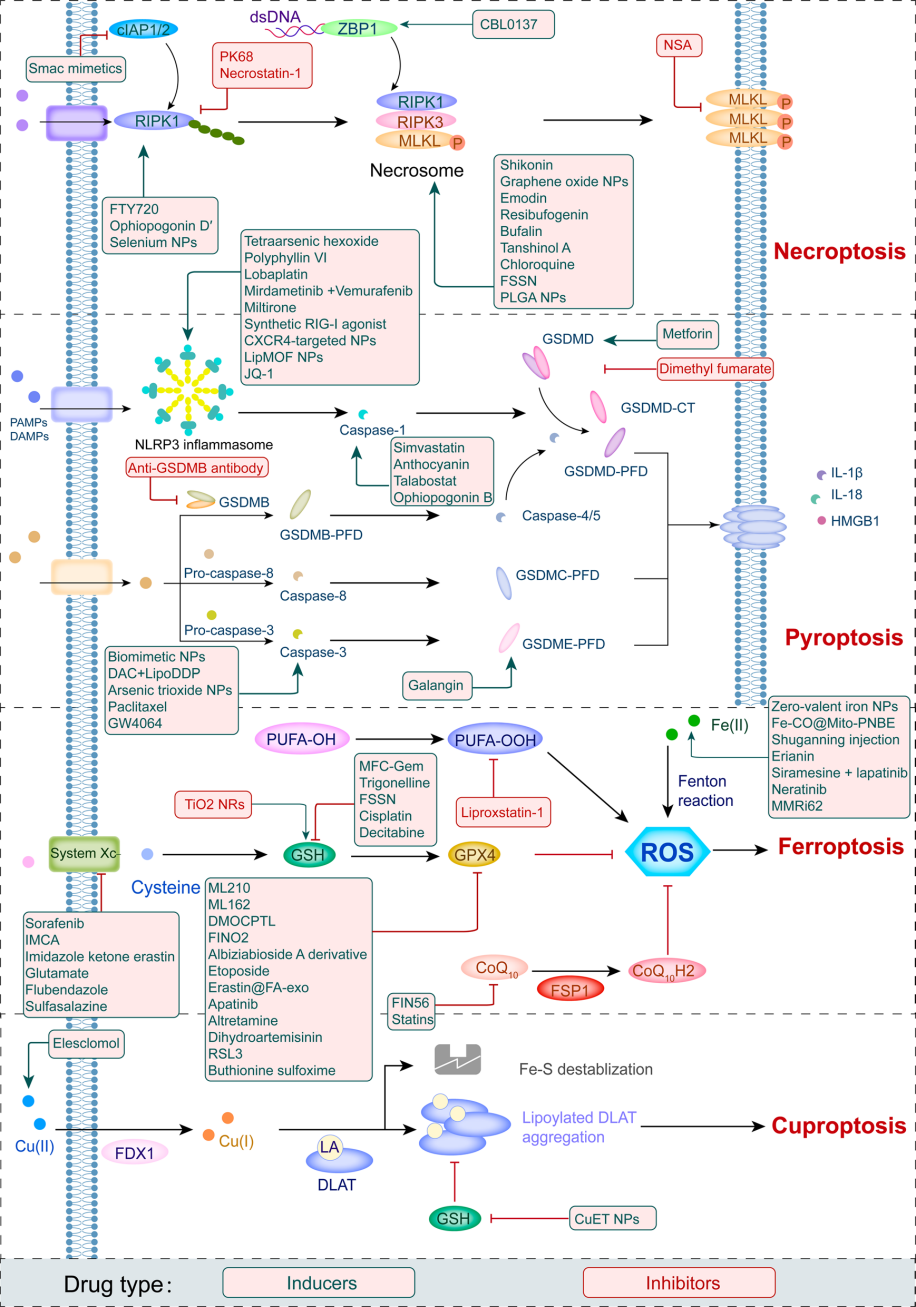
Summary of the modulators of novel RCDs in cancer treatment

In addition, metformin inhibits cancer cell proliferation by inducing mitochondrial dysfunction to cause pyroptotic cell death [241]. Specifically, metformin is a sensitization agent that enhances AMPK/SIRT1/NF-κB signaling to trigger the activation of caspase-3 and the generation of GSDME-PFD. Lu Wang and colleagues documented that metformin causes pyroptotic death of esophageal squamous cell carcinoma cells by targeting the miR-497/PELP1 axis [242]. Further, chemotherapeutic medications, including actinomycin-D, doxorubicin, topotecan, and bleomycin, stimulate the pyroptotic death of GSDME-expressing cells [38]. Teng et al. also found that the induction of ROS/ NLRP3/GSDMD signal axis via using polyphyllin VI practically leads to pyroptotic death of NSCLC cells [246]. Our Fig. 6 summarizes other methods of action of pyroptosis inducers.

Sorafenib is an FDA-approved anticancer drug for the treatment of HCC, RCC, and thyroid cancer [308]. Sorafenib inhibits system X_C_^−^, thus promoting ferroptosis by inhibiting GSH production [281]. Additionally, sorafenib and sulfasalazine may synergize to prevent the activation of branched-chain amino acid aminotransferase, a principal enzyme involved in sulfur-based amino acid metabolism. This therapeutic approach induced ferroptosis in HCC cell lines both in vitro and in vivo [309]. Additionally, cisplatin triggers ferroptosis via GSH depletion and inactivation of GPX4 in NSCLC and colon cancer [197]. Etoposide is a phenolic antitumor drug that efficiently removes GSH in myeloperoxidase–rich myelogenous leukemia cells, thus decreasing GPX4 levels and leading to ferroptosis [279]. In the study by Ma et al., combining the lysosome disruptor siramesine with lapatinib, a tyrosine kinase inhibitor, induced ferroptotic death of breast cancer cells by inhibiting iron transportation and induction of lipid peroxidation [282].

Peter Tsvetkov and colleagues identified that the Cu ionophore elesclomol induces cuproptosis by inducing lethal proteotoxic stress in various types of cancer cells (as shown in Table 1). However, as indicated by Gao Wei and colleagues, elesclomol causes CRC cells to undergo Cu-dependent ferroptosis by promoting the degradation of Cu-transporting ATPase 1 and subsequently inducing ROS accumulation, which promotes the degradation of SLC7A11 [75]. Since current experimental study on cuproptosis is still in its infancy, more research is needed to support its potential for cancer treatment.

##### Nanoparticles (NPs) targeting RCD pathways

Advantages of NPs include easy cell barrier penetration, preferential accumulation in specific organelles and cells, and an increased likelihood of effective fine-tuning, endowing them with great potential as anticancer therapies [310]. As we mentioned above, shikonin shows great potential as an antitumor treatment by inducing necroptosis. However, the clinical application of shikonin has been restricted due to its poor tumor-specific accumulation, low water solubility, short duration in circulating blood, and a high risk for hazardous side effects on normal tissues [311]. Therefore, Feng et al. constructed an FSSN based on the metal-polyphenol coordination of Fe(III) and shikonin, and FSSN showed not only greater water solubility and lower cytotoxicity than shikonin in normal cells but was also integrated with the function of Fe ions. FSSNs effectively reduced the GSH level and induced ferroptosis and necroptosis in mouse breast cancer cell lines [238]. Additionally, the use of graphene oxide NPs in CT26 colon cancer cells successfully induced necroptosis by enhancing the function of RIPK1, RIPK3, and HMGB1 [229]. Similarly, the group of Praveen Sonkusre reported that when treating prostate adenocarcinoma cells with selenium NPs, necroptosis was induced through increased ROS production and TNF and interferon regulatory factor 1 expression [230].

Furthermore, NPs have been used to induce pyroptosis in malignant cells. For example, the biomimetic NP designed by Pengfei Zhao and colleagues consisted of a hydrophobic nucleus composed of indocyanine green and decitabine and a cell membrane shell. Biomimetic NPs induced the accumulation of calcium in the cytoplasm, leading to mitochondrial damage and caspase-3 activation and subsequently inducing GSDME-mediated pyroptosis in 4T1 cell lines [250]. In addition, Kataoka et al. constructed an ROS-responsive nanoreactor based on polyion complex-forming vesicles by introducing thioketal linkers into a covalently cross-linked membrane network. These ROS-responsive NPs shielded glucose oxidase to induce pyroptosis by generating oxidative stress and inducing glucose deprivation [255].

A recent study described the use of an efficient ferroptosis agent, an FePt@MoS2 NP, which induced the release of more than 30% Fe(II) in the TME within 72 h of treatment to accelerate the Fenton reaction and efficiently induce ferroptosis in various cancer cell lines [280]. Analogously, another study showed that zero-valent iron NPs converted Fe(II) to promote the Fenton reaction, which induced mitochondrial lipid peroxidation in oral cancer cells [266]. Furthermore, a positively charged lipophilic nanocarrier (Fe-CO@Mito-PNBE) targeted the negatively charged mitochondrial membrane, and the subsequent release of Fe(III)/Fe(II) ions effectively facilitated the Fenton reaction and ultimately led to cell ferroptosis [267]. More NPs that induce ferroptosis in tumor cells are listed in Table 1.

The administration of NPs reverses cisplatin resistance in cancer cells by inducing cuproptosis. Exogenous platinum is widely presumed to cause drug resistance induced by high concentrations of GSH in cancer cells. According to Lu et al., the diethyldithiocarbamate-Cu complex effectively induces cuproptosis in A549/DDP cell lines by downregulating FDX1 expression. Most of the administered diethyldithiocarbamate-Cu complex maintained a stable chemical structure when mixed with GSH in solvent, suggesting that it potentially combats cisplatin-resistant cancer cells [306]. Accordingly, research into nanomaterials that induce recently discovered RCD pathways is ongoing, and we expect more and better NPs to be clinically used for cancer treatment in the near future.

##### Small molecules targeting novel RCD pathways

An increasing number of small compounds are being tested to target the necroptotic cell death pathway. For instance, Zhou et al. revealed that emodin, an anthraquinone compound purified from various Chinese medicinal herbs, induces necroptosis in glioma cell lines by enhancing TNF/RIPK1/RIPK3 pathway activation and thus inhibits U251 cell proliferation [231]. Additionally, ophiopogonin D′ induces robust necroptosis in prostate cancer cells through RIPK1 activation [232]. Resibufogenin, a small molecule derived from the bufadienolide family of compounds, significantly inhibits the proliferation of CRC cell lines by upregulating RIPK3 expression [104]. These small-molecule compounds still hold a lot of promise to be applied as clinical medicines because of their remarkable ability to cause tumor cells to undergo necroptosis.

Dobrin et al. found that treatment of triple-negative breast cancer cells with ivermectin induces pyroptosis by activating the P2X4/P2X7-gated pannexin-1 channel [259]. Also, based on accumulating evidence, DHA reduces cancer cell viability and proliferation by modulating different cellular responses [312, 313]. For example, Dumont and colleagues proposed that DHA inhibits NLRP3 inflammasome assembly and the JNK signaling pathway in MDSCs, reducing the 5-fluorouracil-induced generation of IL-1 and increasing the anticancer effectiveness of 5-fluorouracil [314]. Yi-Fan Tan and colleagues also revealed that inhibition of BRD4, either through genetic knockdown or the use of the bromodomain inhibitor JQ1, significantly slows the EMT and the cell proliferation rate and leads to caspase-1/GSDMD-mediated pyroptosis in RCC cells [258]. BRD4 is a member of the BET protein family that is involved in the control of epigenetic modifications [315]. Additionally, the thiopyran derivative L61H10 exhibits great antitumor activity by switching apoptosis to pyroptosis in lung cancer cells [316].

Recently, an increasing number of investigations have demonstrated that small-molecule compounds play essential roles in inducing ferroptosis in tumor cells. Zhang et al. discovered that the benzopyran derivative IMCA significantly downregulates SLC7A11 expression and reduces the contents of cysteine and GSH in cells, resulting in lipid ROS accumulation and ferroptosis in human CRC cell lines [268]. In addition, trigonelline is a plant alkaloid that significantly reduces GSH levels, thus induces ferroptosis in head and neck cancer cells [276]. Furthermore, dihydroartemisinin also exerts a robust effect on inhibiting the proliferation and inducing the ferroptosis of leukemia cells [295]. Similarly, Chang et al. found that a marine terpenoid, heteronemin, induces ferroptosis in HCC cells by initiating lipid peroxidation [317]. As shown in the study by Li et al., the small-molecule MMRi62, which targets MDM2-MDM4, induces ferroptosis by degrading mutant p53 and the heavy chain of ferritin and successfully inhibits the metastasis of pancreatic cancer [298]. As we continue our research, we have gradually discovered much promise in the field of pharmaceuticals that induce ferroptosis in tumor cells, and we are eager to see how these medications will be applied in clinical settings.

##### Other methods to target novel cell death mechanisms

Wan et al. documented that radiation therapy (RT) causes tumor cells to release microparticles with broad antitumor effects and thus abrogates immunogenicity primarily via ferroptotic cell death [318]. Mechanistically, radiation causes lipid peroxidation and ferroptotic cell death through three parallel mechanisms at least [198, 319, 320]. First, RT causes lipid peroxidation by producing excess ROS. RT-generated ROS remove electrons from PUFAs, resulting in the formation of PUFA radicals (PUFAs-OH). Then, these unstable carbon-centered radicals quickly react with oxygen molecules to generate lipid peroxyl radicals (PUFA-OO·), which remove H· from other molecules via the Fenton reaction and ultimately generate lipid hydroperoxides (PUFAs-OOH). Second, radiation increases the expression of ACSL4 to support PUFA-phospholipid biosynthesis, although the precise mechanism by which RT increases ACSL4 levels is still unknown [198]. Third, RT induces GSH depletion, which impairs GPX4-mediated ferroptosis defenses and subsequently promotes ferroptosis [63, 320]. Furthermore, disulfiram, a medicine approved to treat alcoholism, was shown to cause lysosomal membrane permeabilization via a ROS-dependent process, leading to ferroptosis and increasing cellular susceptibility to radiation [288].

Additionally, human umbilical cord mesenchymal stem cells (hUCMSCs) were recently identified as a viable cancer therapy option. For example, these cells prevent NSCLC and HCC cells from migrating [321]. Additionally, hUCMSCs show some advantages over other MSCs because they exhibit minimal immunogenicity and can be produced in large numbers. Following the overexpression of NLRP1 and caspase-4, hUCMSCs cause pyroptosis of the MCF-7 breast cancer cell line; however, hUCMSC treatment has little to no effect on the cell cycle [322].

The growth of schwannomas is proposed to be inhibited via a unique approach based on both the introduction of an adeno-associated virus (AAV-1) and treatment with the GSDMD PFD. This combination was created using an AAV-1-based vector encoding the mouse GSDMD N-terminus under the control of the promoter P0, which is unique to Schwann cells. This gene did not cause neurotoxicity to surrounding tissues following an intratumor injection and inhibited the development of the NF2 and HEI-193 schwannoma cell lines through GSDMD-mediated pyroptosis [323]. The intratumor delivery of GSDMD PFD via AAV-1 offers a better level of protection for the nearby normal tissue since it is more selective than typical medication therapy.

#### Agents inhibiting novel cell death pathways

Necroptosis occurs in cancer cells, and the TME is partially protumorigenic because the inflammation underlying necroptosis may trigger tumor development by promoting cell proliferation, genomic instability, angiogenesis, and metastasis [31]. Liu et al. harnessed the MLKL inhibitor NSA to treat a mouse xenograft model, which significantly delayed tumor growth, providing strong evidence of the protumorigenic role of necroptosis [110]. The necroptosis inhibitor necrostatin-1 also helps reduce colitis-associated tumorigenesis in mice [240]. RIPA-56 is a highly potent and metabolically stable inhibitor of RIPK1 that has been employed to treat a mouse model of inflammatory disease and has shown very high selectivity [324]. Another novel RIPK1 inhibitor PK68 which possesses high efficacy and conserved potency among human, mouse, and rat has been reported to effectively inhibit necroptosis and suppress metastasis of both melanoma and lung carcinoma cells in mice [239]. Although the above-mentioned necroptosis inhibitor has not been implemented in cancer patients, the RIPK1 inhibitor, GSK2982772, is currently being tested in phase 2a clinical studies for patients with inflammatory disease [325].

The utilization of pyroptosis inhibitors has significant research promise because of the dual role that pyroptosis plays in cancer. The study in 2019 revealed that delivering the specific anti-GSDMB antibody in biocompatible nanocapsules significantly inhibited the metastasis and drug resistance of HER2 breast cancer cells [264]. In addition, dimethyl fumarate is an inhibitor of pyroptotic cell death that functions by inactivating GSDMD [265]. Recent research by Jun Jacob Hu and colleagues suggests that the use of disulfiram also prevents pyroptosis by preventing the creation of GSDMD pores in a mouse model of inflammation [326]. In addition, Zhang et al. documented that the MLKL inhibitor NSA reverses pyroptosis by suppressing GSDMD oligomerization [327]. The use of these pyroptosis inhibitors in cellular and animal experiments offers great potential for treating patients with certain type of refractory cancers.

Current evidence suggests that ferroptosis induced by doxorubicin (DOX) was proved to contribute to the side effect of cancer therapy, including cardiotoxicity [328]. The DOX cardiomyopathy is caused by the excess free iron released from heme degradation which accumulates on mitochondria. Inhibition of ferroptosis through using ferrostatin-1 and HO-1 antagonist exerts some protective effect against myocardial injury [329]. In addition, the overexpression of ASCL4 also contributes to intestinal injury induced by irradiation therapy. Ji et al. have shown that troglitazone successfully suppresses lipid peroxidation in intestine through inhibiting ASCL4 and inhibited subsequent tissue damage [330]. Further, the novel findings from Soňa Jantová and colleagues demonstrated that the combination of 2,2,6,6, tetramethylpiperidine-N-oxyl (a ferroptosis inhibitor) capped TiO2 nanorods with UV-A light irradiation not only killed MCF-7 cell lines significantly, but also overcame the multidrug resistance [304]. We currently speculate that ferroptosis inhibition might have played a role in this process, but the mechanisms behind it are still blur and need further study. In addition, Dai et al. have found that the DNA damage caused by ferroptosis could facilitate pancreatic tumorigenesis through 8-hydroxy-2′-deoxyguanosine (a major product of oxidative DNA damage)-STING-dependent pathway. And the administration of ferroptosis inhibitor liproxstatin-1 effectively inhibits the pro-tumorigenesis of ferroptosis process [305].

Finally, the use of cuproptosis inhibitors, the most recent kind of cell death, in cancer has yet to be revealed. GSH was found to inhibit cuproptosis in cells, but this can lead to cisplatin resistance in tumor cells [306]. Furthermore, to help visualize the multiple modes of action, we displayed the modulators involved in four RCDs in Fig. 6.

#### Clinical trials targeting novel RCD modalities

Despite the fact that a variety of reports on novel RCD activators and inhibitors have been published lately, clinical trials evaluating the effects of modulators of novel RCDs are still in their infancy. In this section, we summarize the clinical trials to date in primary outcome measures or interventions that have involved the investigation of relevant biomarkers of novel RCD measurement and list them in Table 2.

**Table 2:** Summary of published clinical trials involving modulators of novel forms of RCDs

Trial number	Condition and disease	Drug/Treatment	Number of included patients	Function	Phase
NCT04229992	Colorectal cancer	Magnesium glycinate, Placebo	240 participants	Necroptosis induction	NA
NCT04739618	Metastatic cancer	Keytruda Injectable Product, Yervoy Injectable Product, GM-CSF	32 participants	Necroptosis induction	Phase 2
NCT02965703	Colorectal adenoma	Aspirin	81 participants	Necroptosis induction	Phase 2
NCT03803774	Locally recurrent head and neck squamous cell carcinoma, nasopharyngeal squamous cell carcinoma, sinonasal squamous cell carcinoma	Birinapant, Intensity-Modulated Radiation Therapy	34 participants	Necroptosis induction	Phase 1
NCT03681951	Pancreatic cancer	GSK3145095, GSK3145095 + Pembrolizumab	8 participants	Necroptosis inhibitor	Phase 2
NCT05493800	Oral Mucositis, lymphoma, multiple myeloma hematologic cancer	MIT-001, normal saline	75 participants	Ferroptosis inhibitor	Phase 2

One clinical study aimed to investigate whether the immediate necroptosis induced by the nonablative cryosurgical freezing could be beneficial to the subsequent injection of immunotherapeutic drugs (NCT04739618). This study recruited 32 participants with metastatic solid cancer who are first treated by nonablative cryosurgical freezing and then receive multiplex immunotherapy (including pembrolizumab, ipilimumab, and GM-CSF) and evaluate overall response rate of radiographic changes. In addition, another study posted in 2018 sought for the efficacy of RIPK1 inhibitor GSK3145095 alone and in combination with pembrolizumab included 8 participants. The serious adverse event rate of this study is 50% and it was terminated following an internal review of the company (NCT03681951).

Another phase II clinical study aims to evaluate the efficacy and safety of ferroptosis inhibitor MIT-001 for the prevention of oral mucositis in patients with lymphoma or multiple myeloma receiving conditioning chemotherapy with autologous hematopoietic stem cell transplantation (NCT05493800). This research was launched on August 9, 2022, and we shall keep track of its progress and other messages about the relationship between ferroptosis and inflammatory side effect of cancer therapy.

So far, we have found only the modulators of these two forms of RCDs, necroptosis and ferroptosis, in clinical trials and the results of these cancer therapy methods remain to be discovered. As high-quality articles on cell death modalities continue to emerge, more clinical trials will be conducted with the research purpose stated as understanding these four cell death modalities; therefore, we believe that in the near future, better use of necroptosis, pyroptosis, ferroptosis, pyroptosis, and other mechanisms will optimize anticancer treatments.

### RCD: is it a potential approach to reverse drug resistance in cancer?

#### RCD modulation and chemoresistance

The data from Wang et al. have revealed that the epigenetic repression of RIPK3 allows NSCLC cell lines to escape from necroptosis, which subsequently increases resistance to chemotherapy [331]. Xu Zhao and colleagues successfully used trichothecin to induce necroptosis in chemotherapy resistant cancers. Mechanistically, the expression of RIPK3 was significantly upregulated by the natural secondary metabolite, trichothecin, and then RIPK3 enhanced the phosphorylation of MLKL and also activated the mitochondria energy metabolism and ROS production, leading to a novel strategy to sensitize cancer cells to cisplatin therapy [303]. Thus, it is suggested that the necroptosis pathways and lipid peroxidation can act synergistically and both play crucial roles in overcoming chemoresistance. Intriguingly, the combination of DHA with cisplatin can synergistically induce cytotoxicity against pancreatic ductal adenocarcinoma because DHA induces ferroptosis via promoting GPX4 degradation, ROS production, and ferritin degradation mediated by NCOA4 [332]. In addition, Ophiopogonin B, a bioactive component of traditional Chinese medicine, was reported to have significant impact on inducing pyroptotic cell death of A549 cells, which helps to alleviate the cisplatin resistance [260]. Further, Jing Guo and colleagues also revealed that adding GW4064, a synthetic FXR agonist, to oxaliplatin can significantly limit tumor cell proliferation in vitro, and slow tumor growth in xenograft mouse models. GW4064 effectively enhanced caspase-3/GSDME-mediated pyroptosis of HT-29 and SW620 cells, which increased the chemosensitivity of cells to oxaliplatin [261]. Cuproptosis was also demonstrated to fight against platinum-based chemotherapy resistance. Lu et al. revealed that the killing effect of cisplatin was detoxified by GSH in A549 cells, while the nanomedicine based on Cu(II) (CuET) exhibited GSH-resistant cytotoxicity and efficiently reversed cisplatin resistance [306].

#### RCD modulation and immunotherapy resistance

Nowadays, immunotherapy represented by ICIs has become a major breakthrough in cancer treatment and has achieved considerable success in clinical treatment of some solid tumors [333–335]. However, the use of ICIs is restricted by the lack of tumor-associated antigens, which results in more than two thirds of the patients to not react to ICI-based monotherapy [169]. However, due to the intricate role of the novel RCD modes in TME, we may anticipate that manipulating RCDs may affect the efficacy of ICIs in cancer patients. Emerging evidence has demonstrated that CD8 + T cells inhibit tumor cells via induction of necroptosis, pyroptosis, ferroptosis [169, 336, 337], and possibly cuproptosis [338]. Like what we mentioned, the novel RCDs in TME seriously activate proinflammatory cytokines, infiltration of cytotoxic T cells, and other lymphocytes, which are significant for the sensitivity of various tumors to ICIs [176]. In addition, the release of Gzm B from CAR-T cells activates caspase-3/GSDME-dependent pyroptosis of target cells, which enhances the efficacy of CAR-T cell therapy [181]. Thus, similar to chemotherapy, the immunotherapy may partially function as the inducers of the novel RCD mechanisms, which might provide an immune-based underpinnings for some novel combination therapies.

By creating vaccine viruses that loaded MLKL expression, Hoecke et al. directly delivered the necroptosis mediator MLKL to tumor cells, which successfully promoted necroptotic death and enhanced antitumor immunity. The potent antitumor immunity is attributed to the increased immunity directly against neo-epitopes [339]. Additionally, the RNA editing enzyme ADAR1 has long been known to be a major repressor of Z-type dsRNA (a substrate of ZBP1), and this suppression mechanism results in resistance and poor reactivity to ICIs, while the use of small-molecule drug, CBL0137, directly induces Z-type dsDNA formation in cells and results in activation of ZBP1-depenent necroptosis, which significantly reverses ICIs unresponsiveness of melanoma mouse models [236]. Similarly, the RIPK1-dependent necroptosis is inhibited by the cIAPs that can be antagonized by Smac mimetics and activate necroptotic death pathway in Burkitt’s lymphoma cell lines [237]. Also, in melanoma, the use of Smac mimetics enhances the response to ICIs via directly controlling immune cell (including B cells, MDSCs, DCs, and cytotoxic T cells) [340]. The evidence demonstrates that we might harness necroptotic mechanism in modify the TME to be more prepared for immunotherapy.

Pyroptosis is the main host defensive mechanism, and it boosts the tumor-killing activity of immune cells [181]. Wang et al. found that in the presence of pyroptosis, ICI-based therapies were effective in killing cold tumor cells, which is attributed to the fired up TME caused by pyroptosis-induced inflammation [341]. Analogously, the engineering of multienzyme-mimicking covalent organic frameworks induces pyroptosis and remodel the TME to trigger durable antitumor immunity for αPD-1 checkpoint blockade therapy [342]. However, the potent proinflammatory role of pyroptosis may cause undesirable side effect in immunotherapy. We previously mentioned that pyroptotic cell death that mediated via CAR-T cell therapy can positively enhance the efficacy, but it also counteracts the effectiveness of CAR-T therapy via initiating cytokine release syndrome [343]. The cytokine release syndrome is a severe side effect brought on by an amplified inflammatory reaction mediated by pyroptosis. In detail, IL-1β and IL-18 are released through the first activated Gzm B/caspase-3/GSDME pathway in target tumor cells, which later amplifies the inflammatory response by activating the caspase-1/GSDMD axis in MΦs [343].

It has been reported that lipid peroxides caused by ferroptosis can be utilized as a signal to facilitate the recognition and processing of tumor antigens by DCs and present them to CD8 + T cells, activating cytotoxic T lymphocytes to enhance tumor immunotherapy [344]. Thus, the combination of ferroptosis inducers with ICIs might be an excellent choice for sensitizing malignant cells to immunotherapy. Indeed, the research from Weimin Wang and colleagues has shown that the combination of GPX4 inhibitor, cyst(e)inase with PD-L1 blockade, can improve T cell-induced antitumor immunity and ferroptotic death of cancer cells synergistically [345]. Similarly, an innovative NRF2 nanomodulator, ZVI-NP, which both inhibits the antiferroptotic function of NRF2 and generates massive ROS via Fenton reaction, can potently augment antitumor immune response by reprograming the TME [194]. However, similar to the consequence of macrophage pyroptosis, the ferroptotic death of nontumor cells is associated with impaired antitumor ability because of reduced cytotoxic cytokine production. And harnessing ferroptosis inhibitor ferrostatin-1 significantly prevents CD8 + T lymphocytes ferroptosis via suppressing lipid peroxidation; consequently, cytokine production is increased, resulting in tumor clearance. More importantly, ferroptosis inhibition therapy obtains greater antitumor efficacy when in combination with anti-PD-1 antibodies [346].

Since cuproptosis is a novel mode of cell death reported this year, its role in immunotherapy is more focused on research in bioinformatics. The cuproptosis-related modification patterns developed by Zhiyong Cai and colleagues were demonstrated to be employed in prediction of immune cell infiltration in TME and evaluation of an individual’s sensitivity to ICIs [347]. It is highly likely that cuproptosis can also play a role in tumor immunotherapy, so we look forward to more experimental studies on the aspect of cuproptosis. Based on these findings, we assume that the combination therapy strategies might possess great potential to alleviate the challenge of monotherapy resistance, such as 1. combination of RCD modulators with conventional drug therapy; 2. combination of chemotherapy drugs and immunotherapy; and 3. combination of immunotherapy with radiotherapy.

## Conclusions

Approaches targeting novel RCD modalities hold promise as novel treatments for cancer, and considerable efforts are devoted to translating novel regulators to the clinic. Thus, we complemented the review of approved drugs that modulate novel RCD pathways with descriptions of some newly developed beneficial small-molecule compounds and nanomaterials and clinical trials which intend to explore the changes in the expression levels of four novel biomarkers of RCD. Finally, RCDs can also make tumors more responsive to immunotherapy by regulating tumor immunogenicity and enhancing lymphocyte infiltration in the TME.

However, in spite of the discovery of many compounds and agents that induce or modulate novel RCD programming and that exert strong antitumor effects, many studies reported opposite outcomes. For instance, Chao-Chieh Lin and colleagues discovered that the expression of the key necroptosis mediator RIPK3 in recurrent tumor cells contributed to clonogenic cell growth, causing p53 destabilization and promoting the activities of the YAP/TAZ pathways [348]. Yee et al. also found that ferroptosis induced by neutrophils played a significant role in promoting the aggressiveness of glioblastoma [349]. Therefore, accurate identification of the role of RCD in different types of cancer allows for better utilization of RCD modulators. Greater knowledge of the role played by the TME in controlling tumor cell death may also facilitate the development of cancer eradication therapy. In conclusion, our review postulates that strategies for the pharmacological modulation of novel tumor cell death pathways may be very helpful in cancer treatments, and we encourage future studies using animal models to identify additional outcomes. Additionally, we hope that more clinical trials investigating the use of novel cell death modulations in cancer patients will be conducted.

## Data Availability

The data used to support this study are included within the article.

## Abbreviations

RCD: Regulated cell death
TME: Tumor microenvironment
DAMPs: Damage-associated molecular patterns
PAMPs: Pathogen-associated molecular patterns
MLKL: Mixed-lineage kinase domain-like protein
RIPK1: Receptor-interacting protein kinase 1
RHIM: RIP homology interaction motifs
ZBP1: Z-dsDNA/dsRNA-binding protein 1
GSDM: Gasdermin
PFD: N-terminal pore-forming domain
Gzm B: Granzyme B
CAR-T cells: Chimeric antigen receptor T cells
NK: Natural killer
PUFAs: Polyunsaturated fatty acids
GPX4: Glutathione peroxidase 4
GSH: Glutathione
System Xc^−^: Cystine–glutamate antiporter
ACSL4: Acyl-CoA synthetase long-chain family member 4
ROS: Reactive oxygen species
RCC: Renal cell carcinoma
Cu: Copper
FDX1: Ferredoxin 1
TCA cycle: Tricarboxylic acid cycle
MΦs: Macrophages
GOx@[Cu(tz)]: Glucose oxidase (GOx)-engineered nonporous Cu(I) 1,2,4-triazolate
EMT: Epithelial-to-mesenchymal transition
CRC: Colorectal cancer
NSCLC: Non-small cell lung cancer
CAFs: Cancer-associated fibroblasts
HCC: Hepatocellular carcinoma
MSCs: Mesenchymal stroma/stem cells
ICIs: Immune checkpoint inhibitors
DCs: Dendritic cells
TAMs: Tumor-associated MΦs
MDSCs: Myeloid-derived suppressor cells
BRAFi + MEKi: Combination of BRAF inhibitors and MEK inhibitors
ASAH2: N-acyl-sphingosine amidohydrolase
CQ: Chloroquine
NSA: Necrosulfonamide
TiO2 NRs: 2,2,6,6, Tetramethylpiperidine-*N*-oxyl capped TiO_2_ nanorods
NPs: Nanoparticles
RT: Radiation therapy
HUCMSCs: Human umbilical cord mesenchymal stem cells
DOX: Doxorubicin
CIAPs: Cellular inhibitor of apoptosis proteins
